# A bioactive soluble recombinant mouse LIGHT promotes effective tumor immune cell infiltration delaying tumor growth

**DOI:** 10.1007/s00109-025-02552-x

**Published:** 2025-06-02

**Authors:** Maria-Luisa del Rio, Oscar-Mariano Nuero-Garcia, Giovanna Roncador, Raquel Garcimartín-Bailon, Juan-Carlos Cubria, Pascal Schneider, Jose-Ignacio Rodriguez-Barbosa

**Affiliations:** 1 Transplantation Immunobiology and Immunotherapy Section, Institute of Molecular Biology, Genomics, and Proteomics (INBIOMIC), University of Leon, Leon, 24071 Spain; 2 Molecular Interactions Facility, Department of Cellular and Molecular Biosciences, Centro de Investigaciones Biológicas Margarita Salas, CIB-CSIC, Ramiro de Maeztu 9, 28040 Madrid, Spain; 3 Monoclonal Antibody Unit, Spanish National Cancer Research Centre, Madrid, Spain; 4 Department of Biomedical Sciences, School of Veterinary Medicine, University of Leon, 24071 Leon, Spain; 5 Department of Immunobiology, University of Lausanne, 1066 Epalinges, Switzerland

**Keywords:** HVEM, Herpesvirus entry mediator, LIGHT, LTβR, B16.F10 melanoma

## Abstract

**Abstract:**

The TNF family member LIGHT (TNFSF14) binds to two receptors, HVEM (TNFSFR14) and LTβR (TNFSFR3). HVEM functions as a costimulatory molecule, whereas LTβR is involved in the development of lymph nodes and ectopic tertiary lymphoid structures at chronic inflammation sites. The classical approach of fusing soluble recombinant proteins to the Fc fragment of IgG resulted in a functionally inactive Ig.mouse (m) LIGHT protein. However, in line with the fact that TNF family members cluster receptors as trimers, addition of a small homotrimeric domain (foldon) N-terminal of mLIGHT produced an Ig.Foldon-mLIGHT protein able to bind and engage HVEM and LTβR in a cell-based reporter bioassay. In the tumor model of B16.F10 melanoma cells implanted into syngeneic recipients, cells transduced with membrane-bound mLIGHT grew as aggressively as mock-transduced cells, but growth of tumors of B16.F10 cells expressing Ig.Foldon-mLIGHT was delayed and characterized by significant immune infiltration of dendritic cells and cytotoxic cells. This work unveils the potential of active soluble LIGHT, as a single agent, to recruit cytotoxic cells and dendritic cells at the tumor site to inhibit tumor growth. This effect may be further enhanced with immune checkpoint blockade therapies.

**Key messages:**

- The classical approach of fusing soluble recombinant proteins to the Fc fragment of IgG resulted in a functionally inactive Ig.mouse (m) LIGHT (TNFSF14) protein.
- The addition of a small homotrimeric domain (foldon) N-terminal of mouse LIGHT produces a proper folded bioactive mouse LIGHT recombinant protein.
- Constitutive intratumor expression of secreted Ig-Foldon-LIGHT, but not membrane LIGHT, delays tumor growth.
- Tumors secreting LIGHT, as a single agent, promote beneficial anti-tumor responses through the recruitment and infiltration of cytotoxic cells and dendritic cells.

**Graphical Abstract:**

The intrinsic source of native soluble LIGHT and LTα1β2 (produced by neutrophils and activated T cells and NK cells) along with the tumor secreting recombinant Ig.Foldon-LIGHT co-stimulate NK and T cells through HVEM and license DC for antigen presentation to promote anti-tumor T cell responses in the tumor draining lymph nodes. Furthermore, native LIGHT produced by neutrophils, NK, and T cells and recombinant LIGHT along with LTα1β2 secreted by recruited B cells (LTi, lymphoid tissue inducer cells) would together activate stromal cells of the tumor microenvironment (TME) through LTβR (LTo, lymphoid tissue organizing cells) to release chemokines driving endothelial activation and the upregulation of adhesion molecules that in turn would facilitate transmigration of immune cells from the blood vessels to the tumor site. It would also condition myeloid cells expressing LTβR to secrete cytokines such as TGF-beta that contribute to blood vessel normalization.

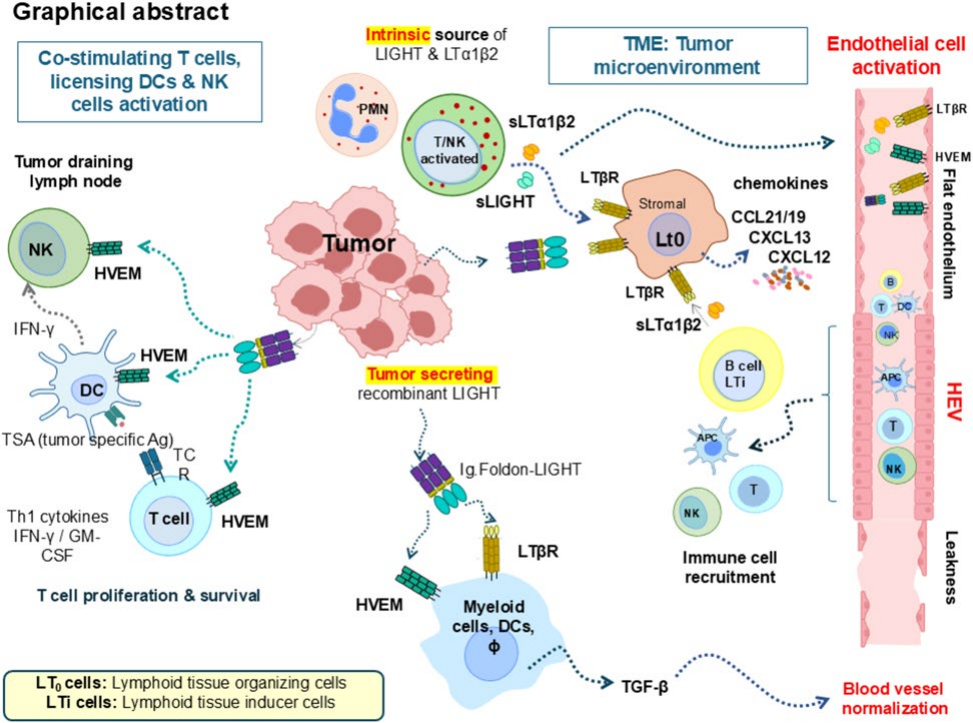

**Supplementary Information:**

The online version contains supplementary material available at 10.1007/s00109-025-02552-x.

## Introduction

The TNF superfamily comprises 19 type II transmembrane proteins with an N-terminal intracellular region and a conserved trimeric C-terminal extracellular domain known as the TNF homology domain (THD) [1]. One of these proteins is LIGHT (homologous to lymphotoxin that exhibits inducible transient expression and competes with herpes simplex virus glycoprotein D for herpes virus entry mediator (HVEM), a receptor expressed by T lymphocytes). The 239 amino acids-long mouse LIGHT (TNFSF14, CD258) is 74% similar to human LIGHT in the THD and regulates innate and adaptive immune responses by engaging two receptors, the herpes virus entry mediator (HVEM, TNFSFR14, CD270) and the lymphotoxin beta receptor (LTβR, TNFSFR3). LIGHT can be either membrane-bound or released as a soluble cytokine by a metalloprotease [2–4].

LIGHT is structurally related to lymphotoxins (LTα3 and LTα1β2) and is transiently expressed on the cell surface of activated T cells, activated natural killer (NK) cells, neutrophils, dendritic cells (DC), and other immune innate cells, but not on naive T cells, regulatory T cells, or B cells [5–7]. HVEM is expressed predominantly in hematopoietic cells, including naive CD4 T cells, CD8 T cells, B cells, myeloid cells, endothelial cells, and in the epithelium of the mucosa at barrier sites [5, 8, 9]. LTβR expression is restricted to endothelial, stromal, and myeloid cells, but is not detected on lymphoid cells [10, 11]. LTα1β2, which also exists as membrane-bound and soluble molecules, is expressed on activated T cells, B cells, and NK cells and its binding to LTβR promotes lymph node development during embryogenesis. In adults, LTβR contributes to the transition of flat endothelium to high endothelial venules (HEV) in pathological scenarios of chronic inflammation. HEV are critical structures for the immune cells to gain access to the tumor microenvironment and for the subsequent formation of tertiary lymphoid structures [12, 13].

The engagement of HVEM by LIGHT in *trans* unleashes CD28-independent costimulatory and survival signals on T cells and NK cells present in the surrounding proinflammatory environment and sustain their differentiation towards effector cells [14–21]. LIGHT and LTα1β2 also bind to LTβR on stromal cells to induce the release of chemokines attracting lymphoid and myeloid cells. LTβR signaling increases adhesion molecules on endothelial cells, bolstering leukocyte attachment and subsequent transmigration that is of particular importance for the recruitment of primed anti-tumor CD8 T cells stimulated in the tumor draining lymph nodes [4, 22].

Unfortunately, the classical approach of fusing recombinant proteins to the Fc fragments of IgG resulted in inactive Ig.mLIGHT proteins [2, 23], hampering studies of potential effects of exogenous mouse LIGHT on the immune response in preclinical models of disease. Here, we describe a method of inserting a small homotrimeric domain between the Fc and LIGHT proteins to obtain Ig.Foldon-mLIGHT that binds HVEM and LTβR. In situ delivery of Ig.Foldon-mLIGHT by B16.F10 melanoma cells, unlike membrane-bound LIGHT, robustly promoted immune cell infiltration and subsequent control of tumor growth.

## Material and methods

### Plasmids for the expression of genes of interest into eukaryotic cells

Table 1 contains a list of plasmids used for the expression of the genetic constructs of interest. The extracellular regions of mouse HVEM or mouse LTβR were fused to the transmembrane and intracellular domains of human Fas and cloned into retroviral pMSCV-pgk-Puro vector. Genetic constructs of mouse LIGHT were prepared for protein expression in HEK-293T cells or for transduction in B16.F10 melanoma cells. Plasmids were produced in TOP10 or Stbl3 strains of *Escherichia coli*. Plasmids were sequenced by Sanger sequencing.

**Table 1:** List of plasmids used for the expression of genetic constructs of interest

Plasmid ID	Designation	Protein encoded	Vector or provider
ps3535	mHVEM:Fas	mHVEM (aa 1–206)-VD-hFas (aa 169–335)	pMSCV
pIB-449	mLTβR:Fas	mLTβR (aa 1–217)-VD-hFas (aa 169–335)	pMSCV
pIB-229	pMSCV-pgk-puro	Modified pMSCV-puro (Clonetech) with Hindlll-Bglll-EcoRl-Notl-Xhol-Hpal-Apal sites	pMSCV
pIB-176	mLIGHT full	mLIGHT (aa 1–239)	pMIG
pIB-113	Ig.mLIGHT	Igk leader-AAQPARRARRTKLGTELGS-mIgG2a (aa 98–330)-DI-mLIGHT (aa 72–239)	pSecTag2
pIB-173	Flag-Foldon-mLIGHT	Preprotrysin leader-Flag-Foldon-mLIGHT (aa 72–239)	pFLAG-CMV3 [23]
pIB-52	mBTLA.Ig	Igk leader-AAQPARRARRTKL-mBTLA (aa 30–184)-GS-mIgG2a.Fc (aa 96–330)	pSecTag2 [59]
pIB-167	Flag-hLIGHT	VCAM1 leader-Flag-YV-hLIGHT (aa 89–240)	Dr. Carl Ware
pIB-459	Ig.Fc-Foldon-mLIGHT	Igk leader-mIgG2a.Fc (aa 98–330)-Foldon-mLIGHT(aa 72–239)	pMSCV
pIB-231	Hit60 MoMuLV (Gag/pol)	Packaging plasmid	other
pIB-232	pCG-VSV-G	Pantropic envelope	other

Igk leader: METDTLLLWVLLLWVPGSTGD

Preprotrysin leader: MSALLILALVGAAVA

VCAM1 leader: MPGKMVVI

HA leader: MAIIYLILLFTAVRG

Flag: DYKDDDDK

Foldon: GYIPEAPRDGQAYVRKDGEWVLLSTFL

*aa* amino acids, *VCAM1* vascular cell adhesion molecule

### Production and purification of recombinant proteins

HEK293T cells were cultured and expanded in adherent 15-cm diameter dishes in DMEM 10% FCS, plus additives (glutamine 2 mM, pyruvate 1 mM, HEPES 10 mM, 1X non-essential amino acids, 0.05 mM 2-βME, gentamycin 50 µg/ml). The day before the transfection, HEK293T cells were trypsinized and seeded at 12 × 10^6^ per 15-cm diameter Petri dishes in 20 ml of complete medium. The day after, the medium was aspirated and 30 µg of plasmid in 1.5 ml of Opti-MEM was mixed with 90 µg polyethylenimine 40 kDa (PEI) transfection reagent (Polysciences, Cat. # 24,765 #) (i.e., 3 µl of PEI (1 mg/ml) per µg of plasmid), left for 20 min, then added to cells. Then, an additional amount of 5.5 ml of Opti-MEM was added to cover the plates. Six hours post-transfection, plates were filled up with 15 ml of Opti-MEM. Five days after transfection, the supernatant was collected, centrifuged, passed through a 0.22-µm filter and purified by affinity chromatography on protein G Sepharose (Biovision) (Ig.mLIGHT, Ig.Foldon-mLIGHT, mBTLA.Ig), whereas Flag-human LIGHT was purified by immunoaffinity chromatography using anti-Flag M2 antibody bound to agarose (Sigma), dialyzed against endotoxin free PBS, filtered through 0.22-µm pore size, and stored at − 80 °C, until use.

### Antibody coupling to NHS-Sepharose and immunoprecipitations

Two milligrams of monoclonal antibody 3D11 anti-mouse LIGHT [6], or anti-human APRIL mAb Aprily2 as an irrelevant control, was coupled to 1 ml of NHS-activated Sepharose beads (GE Healthcare, #90–1004-00). Beads in isopropanol were washed with three-volume column of ice-cold 1 mM HCl, then coupled to 1 ml antibodies at 2 mg/ml in 0.2 M NaHCO3, 0.5 M NaCl, pH 8.3 for 30 min, washed with 3-column volumes of ethanolamine buffer (0.5 M ethanolamine buffer, 0.5 M NaCl, pH 8.3), three-column volumes of acetate buffer (0.1 M sodium acetate, 0.5 M NaCl, pH 4), and again three-column volumes of ethanolamine buffer. After 30 min, beads were washed with PBS, then stored at 4 °C in PBS 0.05% azide until use. Thirty to 50 ml of conditioned cell supernatant were immunoprecipitated for 2 h or more at 4 °C on a rotating wheel with 40 µl of a 50% slurry of beads in PBS. Beads were recovered by centrifugation (3 min at 1500 rpm), transferred in home-made mini-columns (Ref 26 Schneider/Willen/Smulski), washed with 3 × 200 µl of PBS, and eluted with 2 × 30 µl of 50 mM Na-citrate pH2.7 in a tube containing 7 µl of 1.5 M Tris–HCl pH8.8. These proteins were use in cell viability assays with mHVEM:Fas 3534 cl4 reporter cells, or for Western blot and/or for size exclusion chromatography.

### Size exclusion chromatography and protein precipitation

Recombinant mouse LIGHT proteins in a volume of 250 µl were size fractionated at 0.55 ml/min on a Superdex S200 Increase HR 10/30 column (Cytiva) equilibrated in PBS, with online absorbance monitoring at 280 nm and 1 ml fraction collection. The column was calibrated with 100 µl of a mixture of thyroglobulin (669 kDa), ferritin (440 kDa), aldolase (158 kDa), and ribonuclease (13.7 kDa) from GE Healthcare, and bovine serum albumin (67 kDa), ovalbumine (43 kDa), carbonic anhydrase (29 kDa), and aprotinin (6.5 kDa) from Sigma-Aldrich, all at 1.4 mg/ml except ferritin at 0.14 mg/ml.

### Western blot

For Western blot analysis, the required amount of protein in 20 µl was mixed with threefold concentrated SDS-PAGE sample buffer, ± 30 mM dithiothreitol, and heated for 2 min at 94 °C. 500 µl of each fraction from the size exclusion chromatography, was mixed with 2 µl of lysozyme at 10 mg/ml and 45.5 µl of 60% trichloroacetic acid, then precipitated for 10 min on ice. After centrifugation, supernatant was discarded and proteins solubilized by heating for 2 min at 94 °C in 30 µl of SDS-PAGE sample buffer, 30 mM dithiothreitol. Half was loaded on a 10% acrylamide gel. After electrophoresis and transfer to nitrocellulose, membranes were stained with Ponceau Red (0.05% Ponceau S in 3% trichloroacetic acid), rinsed with distilled water, and scanned. Membranes were blocked in PBS 0.5% Tween-20, 4% powdered skimmed milk, then revealed with 1 µg/ml of mouse IgG1 anti-Flag M2 antibody (Sigma, F3165) followed by peroxidase-conjugated goat anti-mouse IgG at 1/8000 (Jackson Immunoresearch 115–035–166) (or with anti-mouse IgG only for samples of Ig.LIGHT). After washing, membranes were revealed with ECL spray reagent (Advansta, K-12049-D50) and imaged with an iBRIGHT FL 1500 imaging system (Thermofisher Scientific).

### Transduction of B16.F10 melanoma cells and Fas-deficient Jurkat cells, and selection of clones

mHVEM:Fas clone 4 reporter cells were generated in Fas-deficient Jurkat JOM2 clone 6 cells as described [24]. For the production of retroviral particles, HEK293 T cells were transfected in 10-cm diameter poly-L-lysine precoated adherent Petri dishes. Ten micrograms of Hit60 MoMuLV (Gag/pol), 1.5 µg of pantropic envelope (pCG-VSV-G), and 10 µg of pMSCV-pgk-Puro plasmid (encoding membrane-bound LIGHT or Ig.Foldon-mLIGHT plasmids) in 450 µl of OptiMEM that were mixed with 64.5 µl of lipofectamine 2000 (Thermofisher) (i.e., 3 µl of lipofectamine per µg of DNA) incubated for 20 min and added to cells (Table 1). Retroviral-containing supernatants were harvested 48 h later, filtered by 0.45 µm, and fresh polybrene (Sigma H9268) was added to a final concentration of 8 µg/ml and mixed. Serial dilutions of supernatants plated in 24-well plates were added to adherent B16.F10 melanoma cells or to Fas-deficient human Jurkat (JOM2 clone 6) cell pellets and then spun down for 5 min at 1.250 rpm and seeded in 24-well plates. Transduced cells were cloned by limiting dilution in the presence of 1 µg/ml of puromycin (AG Scientific) [25]. Isolated clones were selected for stable expression of the gene of interest on the cell membrane of the transduced cells (either mHVEM:hFAS or mLTβR:hFAS) by flow cytometry using a rat anti-mouse HVEM antibody (clone 6 C9) or a rat anti-mouse LTβR antibody (clone 4H8) (Figure S1).

Non-transduced (mock) B16.F10 cells, membrane bound LIGHT-, or Ig.Foldon-LIGHT-transduced B16.F10 melanoma cells used in the in vivo experiments were cloned by limiting dilution and the resulting single colonies were screened by flow cytometry with anti-LIGHT antibody (clone 3D11) to detect cell-bound LIGHT expression, whereas a sandwich ELISA was implemented for the selection of a cell line secreting recombinant Ig.Foldon-mLIGHT.

### ELISA for the quantification of recombinant fusion proteins and for the binding assay of Flag-hLIGHT to mouse LIGHT receptors

For the quantification of Ig.Foldon-mLIGHT in supernatants of transduced melanoma cells, a sandwich ELISA was performed by coating with a goat anti-mouse IgG2a/b polyclonal antibody (2.5 µg/ml, Nordic) to capture soluble recombinant proteins. A biotinylated rat anti-mouse IgG2a monoclonal antibody (clone R19-15, 1 µg/ml, Beckton Dickinson) was used as detector antibody, followed by the addition of horseradish peroxidase-coupled streptavidin SA-HRPO (1/10.000). The OD values obtained at 450 nm were extrapolated to the standard curve of known concentrations of mouse IgG2a for the quantification of the recombinant protein in the supernatant.

To assess the binding specificity of Flag-hLIGHT to mouse LIGHT receptors, an ELISA was performed by coating Maxisorp ELISA plates (Nunc) with serial dilutions of control tags (mIgG2a and hIgG1) or recombinant fusion proteins (mHVEM.IgG2a or mLTβR.hIgG1). Binding of Flag-hLIGHT (at a fixed concentration of 50 ng/well) to the mouse LIGHT receptors was detected using a biotinylated anti-Flag monoclonal antibody (clone M2, Sigma), followed by SA-HRPO. After washing, the 3,3′,5,5′-Tetramethylbenzidine (TMB, Merck) substrate was added, and after color development, the reaction was stopped by adding 2M H2SO4, and absorbance was measured at 450 nm.

### Cell viability assay

mHVEM:hFas reporter cells (40.000 cells per well in a final volume of 100 µl) were exposed to titrated amounts of Ig.mLIGHT, Ig.Foldon-mLIGHT, Flag-hLIGHT or mBTLA.Ig for 16 h at 37 °C, 5% CO_2._ At the end of this period of incubation, 20 µl of a mixture of 0.9 mg/ml phenazine methosulfate (MTS, Sigma) and 2 mg/ml 3-[4,5-dimethylthiazol-2-yl]−5-[3-carboxymethoxyphenyl]2-[4-sulfophenyl]−2H-tetrazolium (PMS, Promega) (1/20, v/v) were added for a further 2 to 8 h. Absorbance was measured at 490 nm.

The cytotoxic effects of serial dilutions of mock B16.F10 cells or membrane mLIGHT expressing B16.F10 effector cells (starting at 20.000 cells/well) were monitored after 24 h coculture with mHVEM:hFas target cells (40.000 cells/well). Non-adherent mHVEM:hFas target cells were collected and analyzed for cell viability by flow cytometry (FSC/SSC) with a gate for cells that remained alive. In addition, supernatants from mock B16.F10 cells or Ig.Foldon-mLIGHT expressing B16.F10 effector cells (1 × 10^6^ cells/ml grown during 24 h) were also tested for their cytotoxic activity over mHVEM:hFas target cells (40.000 cells/well), and cell viability was measured at 490 nm using the MTS/PMS assay. All experiments were performed with mycoplasma-free cells. Cell lines were periodically tested by PCR [26].

### Mice and tumor implantation and monitoring of tumor growth

Eight- to 12-week-old C57BL/6 J female mice (H-2^b^) were bred in our own animal facility from breeding pairs purchased from Janvier laboratories (France).

B16.F10 melanoma cells at passage 8 were a gift of Dr. Tobias Bald (Bonn, Germany). B16.F10 melanoma cell line is a derivative subline of original B16 melanoma that was selected for its preferential metastatic tumor tropism for the lung when injected intravenously [27]. Two × 10^5^ B16.F10 melanoma cells in 100 µl of PBS were implanted subcutaneously following the technique described by Allard et al. [28]. Most tumors exhibit an ellipsoid shape. Tumor volume was monitored every 2 days from day 8 to day 16 using a digital caliper and tumor volume was calculated with the following formula: V = (L × W^2^)/2, where length was the greatest longitudinal diameter and width was the greatest transversal diameter.

### Isolation of tumor infiltrating cells

For the isolation of the immune cell infiltrates, tumors were dissected carefully to prevent cross-contamination with brachial and axillar lymph nodes. Dissected tumors were excised finely and digested in 5 ml of digestion buffer HBSS Ca^++^/Mg^++^ without phenol red (containing 5 mg of collagenase D/gr of tumor, 0.5 mg of DNAse I/gr of tumor and supplemented with 25 mM of HEPES pH 7.3, 5 mM CaCl_2_, 5 mM MgCl_2_, and 2% of complement free FCS) and incubated at 37 °C for 20 min under shaking at 200 rpm.

Digested tissue was then passed through a nylon mesh holder of 70 microns to a 50 ml tube and spun down. After a washing step with 7 ml of complete RPMI 1640 medium, the pellet was resuspended into 3 ml of Percoll 40% and layered on top of 3 ml of Percoll 80%, and the interphase was collected after centrifugation at 2000 rpm for 20 min at RT without break. The isolated cells were washed, resuspended in complete RPMI 1640 medium, and spun down. Red blood cells were lysed for 3 min in ACK lysis buffer, cells were washed again, and the pellet was resuspended in 1 ml FACS buffer. One hundred microliters of the cell suspension was incubated with 2 µg/ml of FcR blocker (clone 2.4G2) and then stained with FITC-labeled anti-CD45.2 antibody and the total number of tumor-infiltrating cells was counted in the flow cytometer. The cell counts for that volume were then extrapolated to the final volume of 1 ml in which tumor cells were initially resuspended.

### Flow cytometry

Jurkat cells were collected and resuspended in FACS buffer (PBS containing 2% FCS and 0.5 mM EDTA). Purified Ig.mLIGHT, Ig.Foldon-mLIGHT, Flag-hLIGHT, or mBTLA.Ig fusion proteins (1 µg/ml) were added to the mHVEM:hFas- or LTβR:hFas-deficient Jurkat cells and incubated at 37 °C for 1 h. Cells were then washed in FACS buffer and incubated with biotinylated anti-mIgG2a (R19-15) or biotinylated anti-Flag (M2) mAbs for 30 min at room temperature. After a washing step, the reactions were developed using SA-Alexa Fluor 647 and the samples were acquired by flow cytometry and collected data analyzed with FlowJo V10 software.

The phenotypic analysis of the tumor-infiltrating leukocytes was performed by flow cytometry using the set of monoclonal antibodies conjugated to fluorochromes listed in Table 2. Tumor-infiltrating immune cells were first gated on CD45.2-positive cells (pan leukocyte marker), and from there using successive sub-gating strategies, the different immune cell populations were phenotypically characterized. The binding of biotinylated antibodies to cell surface was revealed with streptavidin (SA)-PE, SA-PE/Cy7, SA-Alexa 647, or SA-BV421, depending on the combination of fluorochromes used in each staining. Dead cells and debris were systematically excluded from the acquisition gate by adding propidium iodide (PI). Live cells were gated as PI-negative and cellular aggregates were excluded according to FSC-H/FSC-A dot plot profile. Flow cytometry acquisition was conducted on a Cytek® Aurora Spectral Cytometer and data analysis was performed using FlowJo software version 10.

**Table 2:** List of antibodies: specificity, biotin/fluorochrome labeling, clone name, and the provider

Target	Labeling	Clone	Company
Lineage-restricted cell surface markers			
Isotype rat IgG_2a_	Biotin	AFRC MAC 157	Home-made (ECACC)
Isotype mouse IgG2b	Biotin	MPC-11	Home made (ECACC)
Mouse IgG2a/2b	Unlabeled	Polyclonal goat anti-mouse anti-mouse IgG2a/b (capture)	Nordic-MUBIO # GAM-Ig-G2a/b
Mouse IgG2a	Biotin	R19-15 (detector)	BD (#553388)
Anti-mouse Ig	HRPO	Polyclonal goat anti-mouse IgG	(Jackson Immunoresearch 115–035–166)
Flag peptide	Biotin	Clone M2	Sigma (#F9291)
FcγR blocker	Unlabeled	2.4G2	Home-made [60]
CD45.2	FITC	104	Biolegend #109806
CD11b	PerCP-Cy5.5	M1/70	BD, #561114
CD11c	APC	N418	Biolegend #117310
CD3	BV711	17 A2	Biolegend, #100241
CD4	PE-Cy7	GK1.5	Biolegend, # 100422
CD8α	PE	53–6.7	Biolegend, # 100708
CD8	BV510	53–6.7	Biolegend, #100752
CD49b	APC	DX5	Biolegend, #108910
LIGHT/HVEM/LTβR/pathway			
LIGHT	Biotin	3D11	Home-made [6]
LIGHT	Biotin	6H12	Home-made
HVEM	Biotin	6 C9	Home-made [8]
LTβR	Biotin	4H8	Gift from Dr. Carl Ware
Co-inhibitory molecules			
PD-1	BV421	29 F.1 A12	Biolegend, #135217

*ECACC* European Collection of Authenticated Cell Cultures

### Statistical analysis

Sample normality distribution was analyzed using D’Agostino and Pearson tests. One way ANOVA and a post-analysis based on Tukey’s test were applied to compare the differences of means among groups. The kinetics of tumor growth volume over time for each experimental group was compared using the correction of Holm-Šídák method, which is a statistical approach used to compare multiple pairs of means at different time points. The statistical analysis was performed using GraphPad Prism 8.0 software (GraphPad Software, Inc).

## Results

### Ig-tagged mouse LIGHT recombinant proteins are unstable and hard-to-purify but can display biological activity

While most TNF family ligands can be produced as active recombinant protein in fusion with the Fc portion of an IgG (Figure S2), this approach does not work in the particular case of mouse LIGHT [2]. This was confirmed using mHVEM:Fas reporter cells that die through the surrogate Fas-mediated death pathway upon mHVEM engagement by an active ligand. No activity could be detected with reporter cells in Ig-mLIGHT-containing conditioned cell supernatants (Figure S3A), while inserting of a trimerization domain (Foldon) at the N-terminus of mLIGHT yielded a protein with detectable activity in assays with the produced supernatant (Figure S3B). This activity was unstable and disappeared with increasing transfection times (Figure S3B). In contrast to Ig.Foldon-mLIGHT, Flag-Foldon-mLIGHT displayed higher activity on reporter cells (especially in the presence of an anti-Flag cross-linking antibody, as shown before for various TNF family members) [29]. This activity could be concentrated, and even partially depleted after affinity purification on a monoclonal anti-mLIGHT antibody (3D11) (Figure S3C). This activity was recovered after elution of 3D11 beads, but with a yield two to three orders of magnitude lower than expected (Figure S3D). The same was observed for Ig-Foldon-mLIGHT: the weak activity present in cell supernatant could be depleted by affinity purification on either 3D11 or protein G, but very little if any activity was recovered in these eluates (Figure S3E, F). Ig-mLIGHT-containing supernatants displayed no or very little activity before and after affinity purification (Figure S3G, H). The target proteins were however present in these supernatants as witnessed by SDS-PAGE, Ponceau red staining (to visualize all proteins), and detection of the mouse Ig portion (for Ig-mLIGHT and Ig-Foldon-mLIGHT) or of the Flag tag (for Flag-Foldon-mLIGHT). They displayed the expected monomeric sizes under reducing conditions (a doublet around 28 kDa for Flag.Foldon-mLIGHT, for a theoric mass of 22.5 kDa plus two N-linked glycans, and a doublet around 55 kDa for Ig-mLIGHT and Ig.Foldon-mLIGHT, for theoretical masses of 47 and 50 kDa plus three N-linked glycans. One N-linked glycan usually has an apparent molecular weight of 2.5 kDa by SDS-PAGE, and at the expected size of a denatured dimer under non-reducing conditions for the Ig-tagged molecules (Figure S4). As expected, proteins were captured by specific affinity purifications, but not by affinity purification on an irrelevant antibody (ctrl, Figure S4). Finally, the analysis of the native size of these purified proteins (and also unpurified Ig-tagged proteins in concentrated cell supernatants) by size-exclusion chromatography revealed migration of the Ig.Foldon-mLIGHT protein and its activity in the higher molecular weight fractions (around 500 kDa) (Figure S5A–B), while Ig-mLIGHT eluted as a double peak (at around 500 and 200 kDa), both devoid of activity, before and after affinity purification (Figure S5C–D). Curiously, the majority of affinity-purified Flag.Foldon-mLIGHT migrated as poorly active high molecular weight species (around 500 kDa), with a much smaller peak of an active species (around 80 kDa, a size compatible with a trimer) (Figure S5E). Taken together, these results confirm the difficulty of expressing active recombinant mouse LIGHT, but point to a beneficial action of the Foldon trimerization sequence to recover activity in Flag-Foldon-mLIGHT, and to a lesser extent in Ig.Foldon-mLIGHT. Even with a Foldon sequence, Ig-mLIGHT remains prone to inactivation, resulting in a low specific activity, probably because of intrinsic instability and/or exquisite sensitivity to acidic conditions.

### The foldon sequence in purified Ig.Foldon-mLIGHT increases or restores binding to receptors

**Fig.1:**
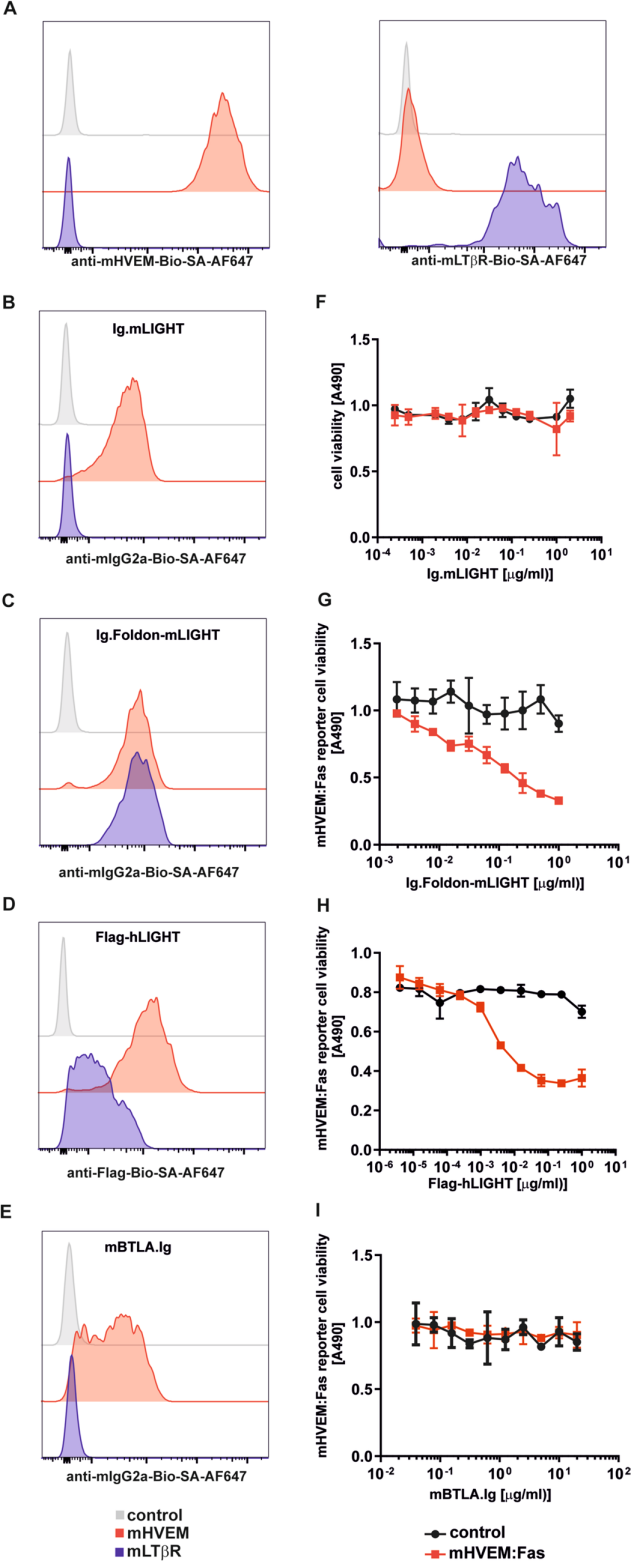
Ig.Foldon-mLIGHT binds mHVEM and mLTbR and activates mHVEM:Fas reporter cells, while Ig.mLIGHT does not exhibit functional activity. mHVEM:Fas (red lines), mLTβR:Fas (blue lines), and control parental Jurkat cells (grey lines) were stained with biotinylated anti-HVEM (**A**, upper panel) or anti-LTβR (**A**, lower panel) monoclonal antibodies. mHVEM:Fas (red lines), mLTbR:Fas (blue lines), and control parental Jurkat cells (grey lines) were stained with recombinant Ig.mLIGHT (**B**), Ig.Foldon-mLIGHT (**C**), Flag-hLIGHT (**D**), or mBTLA.Ig (**E**). The binding of the fusion protein to cells was revealed by the addition of a biotinylated anti-mIgG2a (**B**, **C**, **E**) or biotinylated anti-Flag antibody (**D**), followed by streptavidin-Alexa Fluor 647. This experiment was performed twice. mHVEM:Fas reporter cells (red squares) and parental Jurkat JOM2 cells (black circles) were exposed overnight to serial dilutions of recombinant Ig.mLIGHT (**F**), Ig.Foldon-mLIGHT (**G**), Flag-hLIGHT (**H**), or mBTLA.Ig **(I**). Cell viability was then measured with MTS/PMS colorimetric test. A representative experiment of two performed is shown

Flag-human LIGHT, a protein whose production is straightforward, bound to both mouse HVEM and mouse LTβR proteins by ELISA, validating the folding of these recombinant mouse receptors (Figure S6). Ig-mLIGHT that lacked activity on mHVEM:Fas reporter cells nevertheless bound to mHVEM, but not to mLTβR in a FACS-based assay (Fig. 1A–C, F), while Ig.Foldon-mLIGHT bound both mHVEM and mLTbR in the FACS-based assay and displayed activity on mHVEM:Fas reporter cells (Fig. 1C, G), but with a specific activity much lower than that of the stable Flag-hLIGHT (Fig. 1D, H). Mouse BTLA, a binding partner of HVEM, specifically bound to mHVEM in the form of a mBTLA.Ig but displayed no activity on reporter cells (Fig. 3E, I), even when cross-linked via its Fc portion (by a biotinylated polyclonal antibody captured by plate-bound streptavidin, data not shown).

**Fig. 2:**
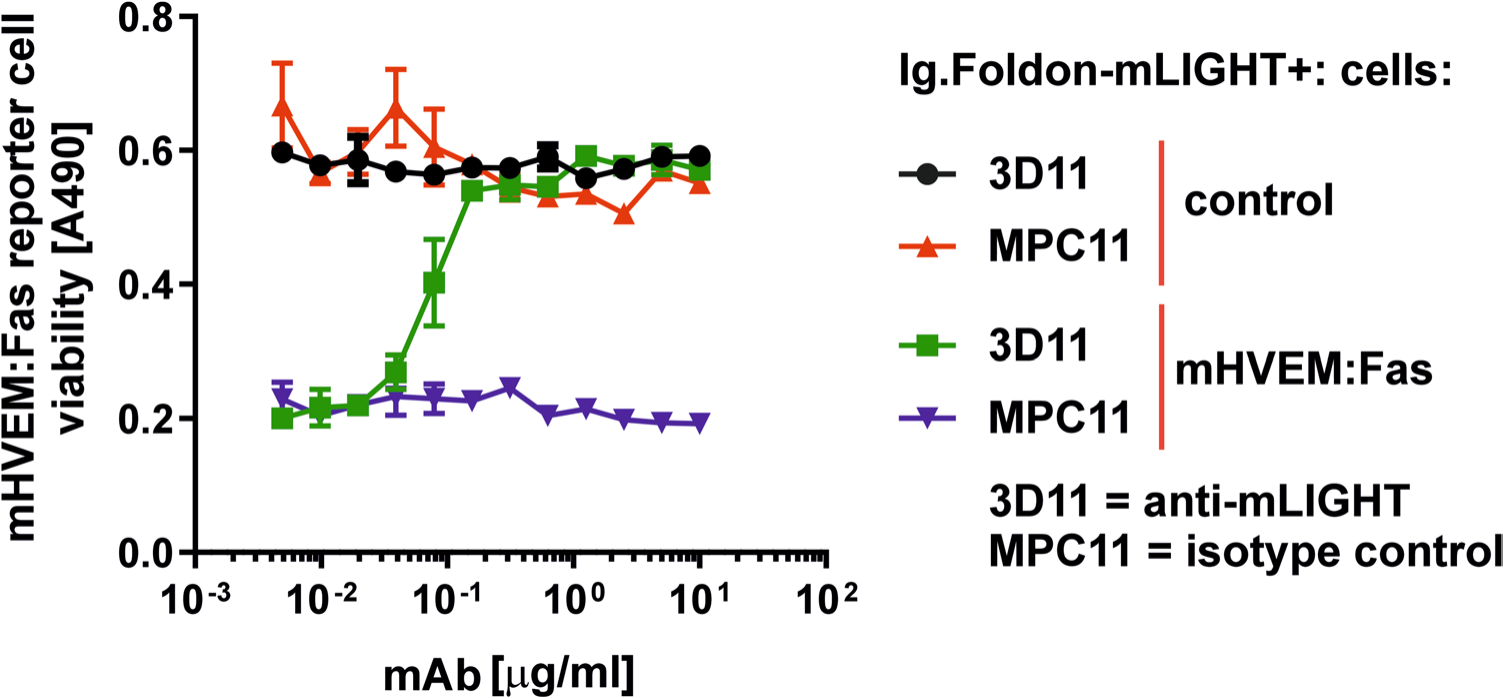
Killing of mHVEM:Fas reporter cells by Ig.Foldon-mLIGHT is blocked by an anti-mLIGHT antibody. mHVEM:Fas reporter cells, or parental Jurkat JOM2 cells used as control, were incubated with a fixed, lethal concentration of 1 µg/ml of Ig.Foldon-mLIGHT preincubated with titrated amounts of an anti-mLIGHT monoclonal antibody (clone 3D11, mouse IgG2b) or of a control antibody (clone MPC11, IgG2b). After an overnight incubation, cell viability was measured with MTS/PMS colorimetric test. A representative experiment of two performed in shown

The specificity of the assay was further validated by preincubation of Ig.Foldon-mLIGHT with 3D11, the anti-mouse LIGHT blocking antibody [6], that inhibited killing of reporter cells by Ig.Foldon-mLIGHT in a dose-dependent manner (Fig. 2).

### Superior therapeutic potential of soluble Ig.Foldon-mLIGHT recombinant protein over membrane-bound LIGHT when transduced into B16.F10 melanoma cells

**Fig. 3:**
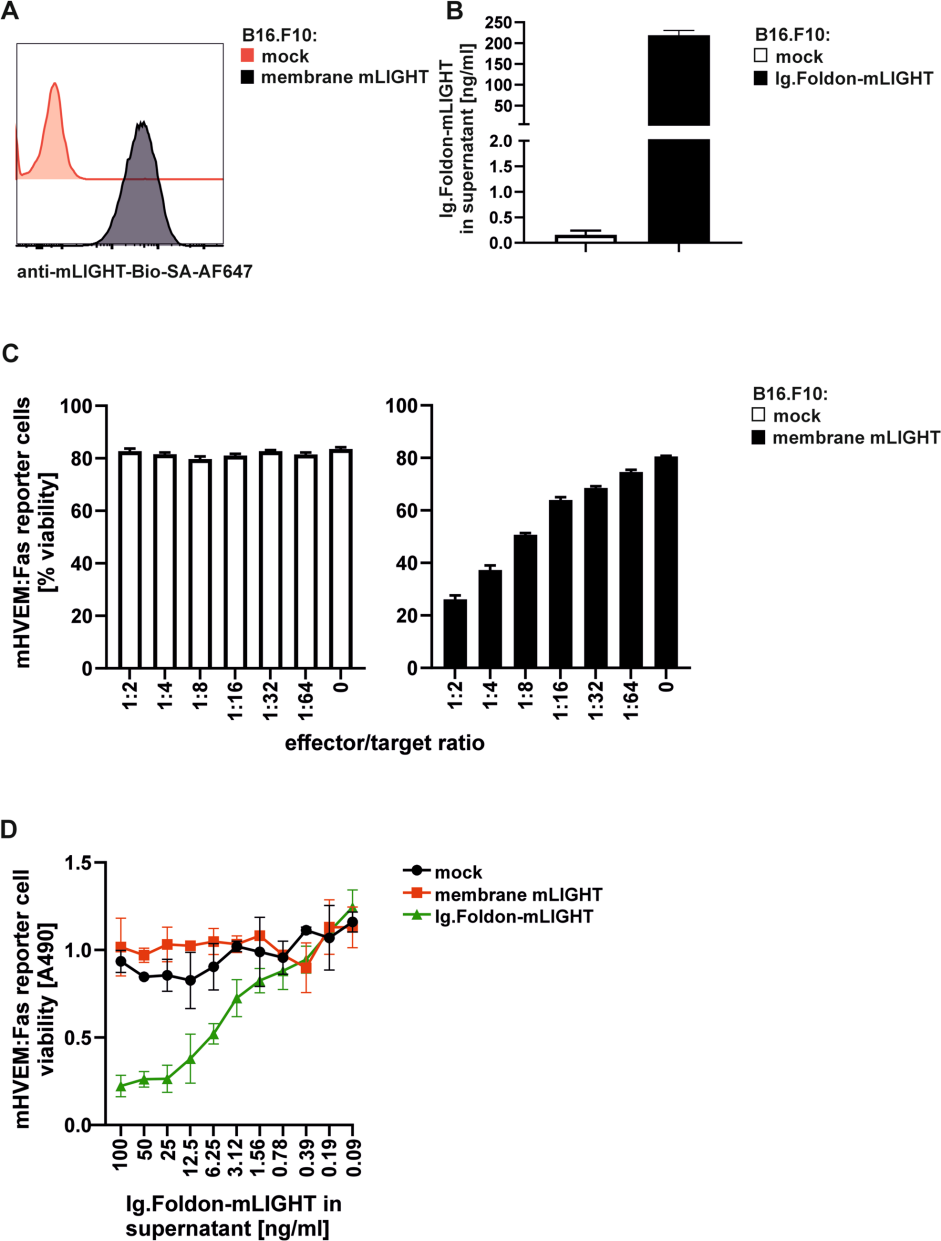
Membrane bound mLIGHT can activate mHVEM:Fas reporter cells. **A** Cell surface expression of full-length mLIGHT expressed in a clone of B16.F10 cells (black line) was revealed by flow cytometry after staining with biotinylated anti-mLIGHT antibody (clone 3D11) followed by streptavidin Alexa Fluor 647. Mock control B16.F10 cells (red line) were used as control. **B** Sandwich ELISA with a pair of anti-mouse IgG2a antibodies was used for the quantification of recombinant protein secreted by B16.F10 melanoma cells. **C** mHVEM:Fas reporter cells (target) were co-cultured overnight with untransduced B16.F10 cells (left panel) or with a clone of B16.F10 cells expressing membrane mLIGHT (effector) at the indicated effector/target ratio (right panel). Cell viability of non-adherent target cells was measured by flow cytometry. Bars represent mean ± SD of triplicates. One representative experiment of two performed is depicted. **D** mHVEM:Fas reporter cells (target) were co-cultured overnight with supernatants from mock B16.F10 cells, membrane mLIGHT B16.F10 cells, or Ig.Foldon-mLIGHT expressing B16.F10 effector cells (1 × 10^6^ cells/ml grown during 24 h). The viability of target cells was measured using the MTS/PMS assay. Symbols mean ± SD of duplicate. The experiment was performed twice with similar results

To assess whether mLIGHT produced at a tumor site may influence tumor growth, tumor cells were directly transduced with mLIGHT. To further delineate the site at which mLIGHT would exert activity, we chose to express the natural membrane-bound form of mLIGHT, presumably active locally, or a soluble form of mLIGHT tagged with an Fc portion to increase half-life in vivo and/or activation of Fc receptors, with an expected longer action range. In view of the difficulty in obtaining active recombinant Ig.mLIGHT in vitro, Ig.Foldon-mLIGHT was selected for this purpose. We first isolated a clone (named pIB-176-1 F8-5) of B16.F10 melanoma cells transduced with membrane-bound mLIGHT by flow cytometry screening using a biotinylated mouse anti-mouse LIGHT monoclonal antibody (clone 3D11) [6] (Fig. 3A). Another high expresser clone (named pIB-459b-1B12) secreting soluble Ig.Foldon-mLIGHT was also isolated after transduction of B16.F10 melanoma cells and subsequent cloning by limiting dilution and screening of individual clones by sandwich ELISA. The amount secreted by the selected clone produced 219 ± 9 ng/ml in a period 24 h after seeding 1 × 10^6^ cells/ml and was quantified by sandwich ELISA using anti-mIgG2a antibodies for capture and detection of the recombinant protein (Fig. 3B).

Mock control B16.F10 melanoma cells did not show cytotoxic activity in the cell reporter bioassay (Fig. 3C, left panel), whereas membrane-bound LIGHT expressed on B16.F10 melanoma cells was functionally active because these cells killed co-cultured mHVEM:Fas reporter cells in an effector to target ratio-dependent manner (Fig. 3C, right panel). However, conditioned cell supernatants of these cells did not kill reporter cells, indicating that membrane mLIGHT B16.F10 cells do not release soluble mLIGHT, or at least not in an active form. In contrast, conditioned supernatants of B16.F10 cells expressing Ig.Foldon-mLIGHT readily killed reporter cells in a dose-dependent manner, with an EC50 of 1.9 ng/ml (Fig. 3D), which was comparable to that of purified recombinant Flag-hLIGHT and better than that of purified Ig.Foldon-mLIGHT (Fig. 2).

**Fig. 4:**
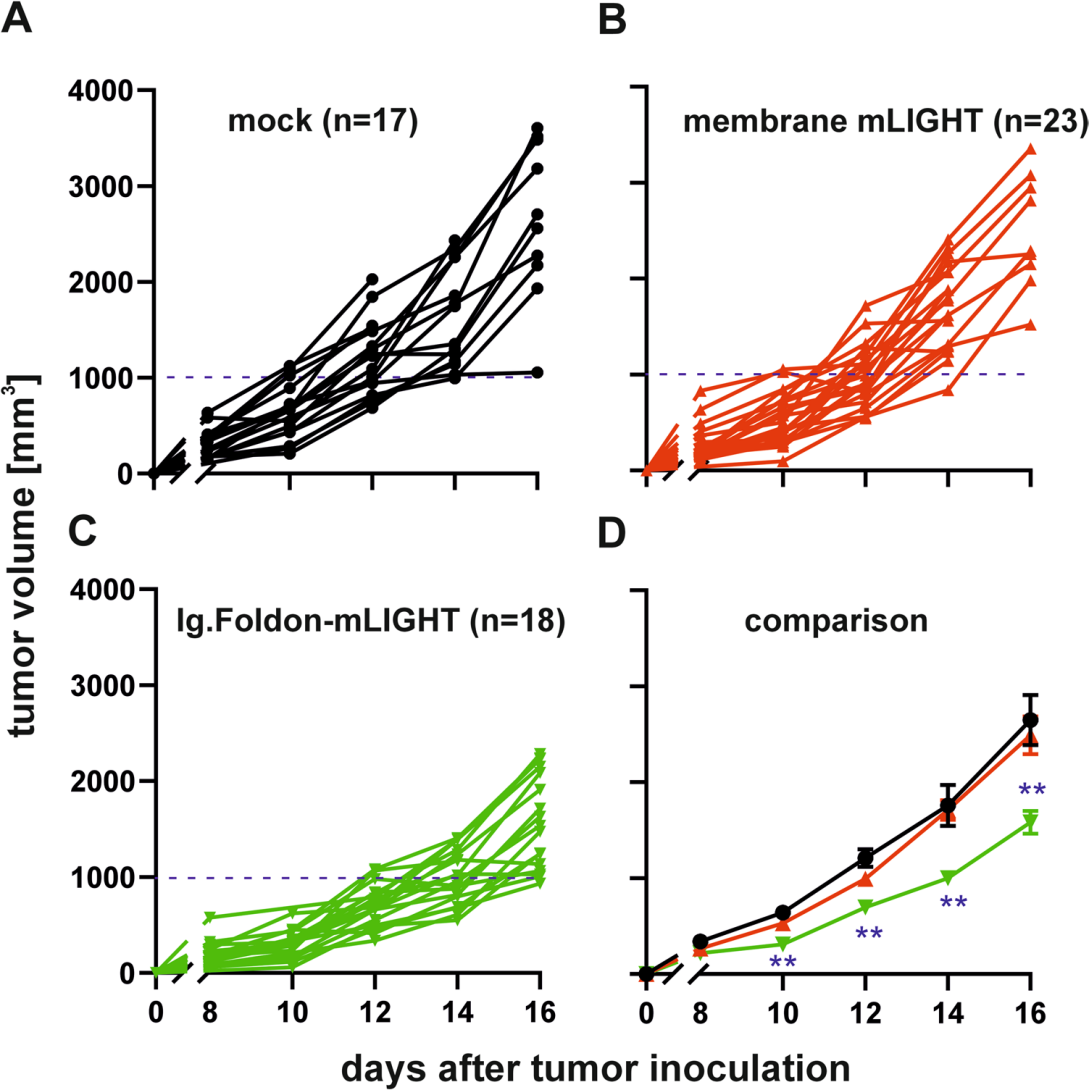
Delayed tumor growth in recipient mice implanted with Ig.Foldon-mLIGHT B16.F10 tumor cells. C57BL/6 mice were injected s.c. with syngeneic mock B16.F10 melanoma cells (*n* = 17) (**A**), B16.F10 expressing membrane mLIGHT (*n* = 23) (**B**), or B16.F10 cells secreting Ig.Foldon-mLIGHT (*n* = 18) (**C**). Tumor volume was recorded every other day from day 8 (visible tumors) to day 16. The average tumor volume over time for all three groups ± SEM are compared by multiple testing for each time point with Holm-Sidak correction. * *p* < 0.01, ** *p* < 0.001, *** *p* < 0.001 (**D)**. These plots are a summary of three independent experiments performed

We next explored whether expression of membrane-bound LIGHT or soluble Ig.Foldon-mLIGHT by tumor cells could modulate tumor growth. Mock control or LIGHT-expressing B16.F10 melanoma cells were implanted into syngeneic mouse recipients and tumor volumes monitored over time until day 16 post-implantation. There was no difference in tumor growth between control and membrane LIGHT-expressing melanoma cells, even though we hypothesized that biologically active membrane LIGHT on tumor cells could stimulate immune and stromal cells in close contact to the tumor (Fig. 4A, B, D). In contrast, tumor growth of melanoma cells expressing Ig.Foldon-mLIGHT was significantly attenuated at multiple time points post-implantation (days 10, 12, 14, and 16) (Fig. 4C, D).

Overall, these observations suggest a model in which active Ig-tagged soluble recombinant mLIGHT would act in a paracrine fashion on HVEM expressed on innate and adaptive immune cells- and/or on LTβR-expressing stromal, endothelial, and myeloid cells at a distance from the tumor site to trigger a local inflammatory response capable of interfering with tumor growth.

### Delayed tumor growth was associated with increased immune cell infiltration into the tumor microenvironment of Ig.Foldon-mLIGHT B16.F10 tumors

**Fig. 5:**
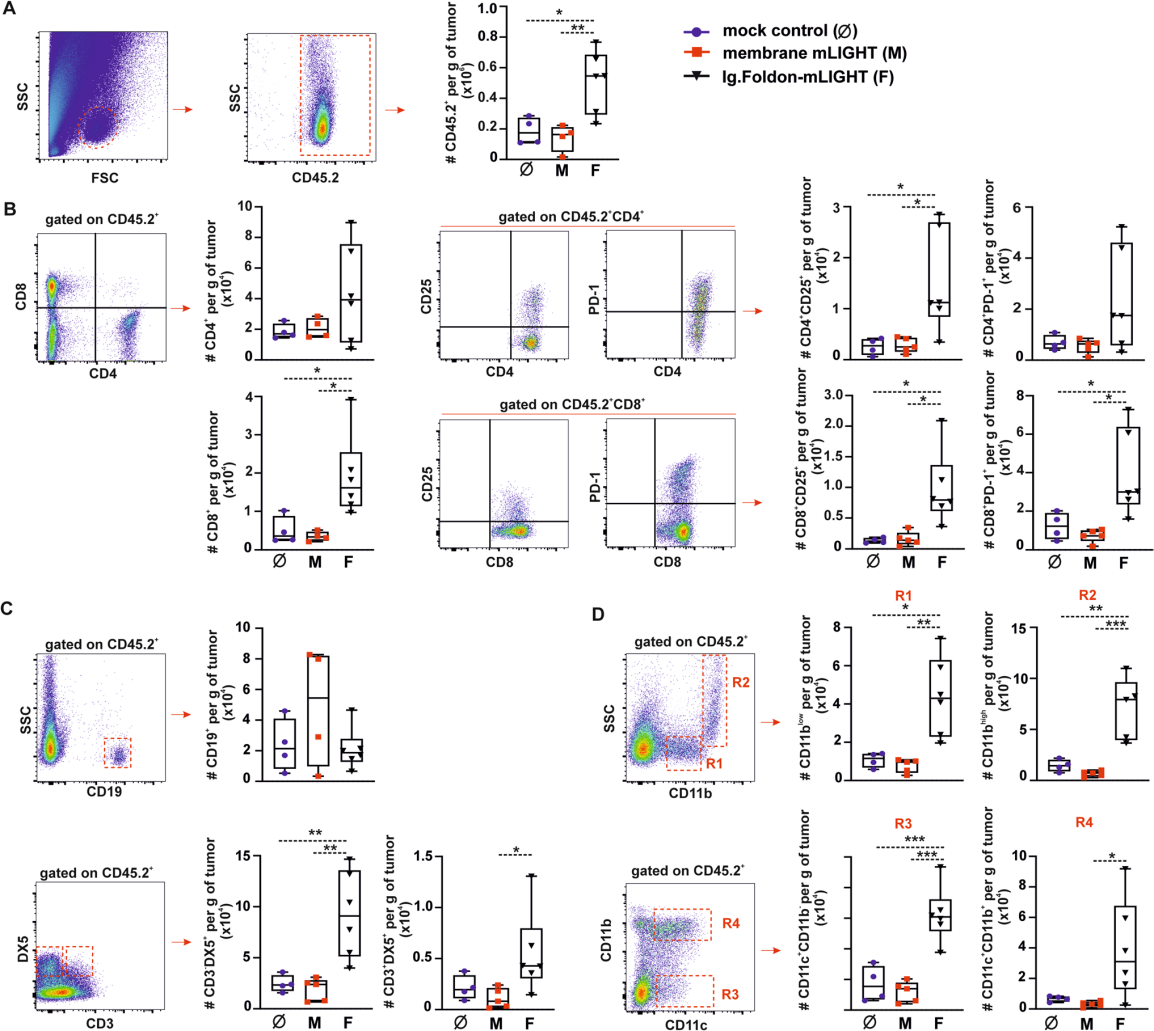
Delayed tumor growth in syngeneic mice implanted with B16.F10 melanoma cells secreting Ig-Foldon-mLIGHT is associated with significant cytotoxic cells and dendritic cells recruitment into the tumor. Tumors of mock control (blue circle, Ø) (*n* = 5), membrane mLIGHT- (red squares, M) (*n* = 5), or Ig.Foldon-mLIGHT-expressing B16.F10 melanoma cells (black triangles, F) (*n* = 6) were resected form syngeneic C57BL/6 mice and used to isolate infiltrated immune cells for phenotypic characterization by flow cytometry. **A** FSC/SSC dot plots were used to gate the population of leukocytes. CD45.2^+^ hematopoietic cells were distinguished from CD45.2^−^ non-hematopoietic stromal cells and enumerated. **B** CD4 and CD8 T cells and their subsets of PD-1^+^ and CD25^+^ cells were identified and counted (as number of cells per gram of tumor) by flow cytometry. **C** Same as panel **B** but for CD19^+^ B cells, CD3^−^/DX5^+^ NK cells, and CD3^+^/DX5^+^ NKT cells. **D** Same as panel **B** but for SSC^low^/CD11b^low^ monocyte-like cells (R1), SSC^high^/CD11b^high^ granulocyte-like cells (R2), and CD11c^+^/CD11b^−^ (R3) and CD11c^+^/CD11b^+^ (R4) dendritic cells. One way ANOVA and a post-analysis based on Tukey’s test was used for the comparisons of means. * *p* < 0.05, ** *p* < 0.005, *** *p* < 0.0005. These plots are a summary of two independent experiments performed

B16.F10 transplantable melanoma is an aggressive tumor model that progresses very quickly over a couple of weeks. Single genetic modifications to express cytokines like Flt3L, GM-CSF, or IP-10 usually do not show any significant anti-tumor activity by themselves unless combined with immune checkpoint blockade or preimmunization strategies with the genetically modified irradiated metabolically active tumor cells before being exposed to live tumor cells [30–32]. This is because B16 melanoma is a poorly immunogenic cold tumor with low expression of MHC class I and with a low rate of tumor immune cell infiltrates. To deepen into the mechanism behind the finding of delayed tumor growth in mice receiving B16.F10 cells expressing Ig.Foldon-mLIGHT, tumor-infiltrating immune cells were counted and phenotypically characterized. There were significantly higher absolute numbers of tumor-infiltrating CD45.2 cells in the Ig.Foldon-mLIGHT group than in the mock control or membrane LIGHT groups (* *p* < 0.05 and ** *p* < 0.005, respectively) (Fig. 5A).

We then compared CD4 and CD8 T cells co-expressing either CD25 or PD-1, NK cells (CD3^−^/DX5^+^), NKT cells (CD3^+^/DX5^+^), and B cells (CD19 cells) in the different tumor groups (Fig. 5B). In general, the absolute number of immune infiltrating cells was higher in the group of Ig.Foldon-mLIGHT B16.F10 tumors than in the others, with the greatest differences seen in comparison with the group of membrane-bound LIGHT tumors that tended to have less infiltrate than mock control tumors (Fig. 5B). CD8 T cells, including CD8^+^/CD25 (IL-2R)^+^ and CD8^+^/PD-1^+^ T cells, CD4^+^/CD25^+^ T cells, NK cells, and NKT cells were all significantly higher in the Ig.Foldon-mLIGHT group than in the membrane LIGHT group (and than in the mock control group, except for NKT cells whose increase did not reach significance). There were no statistical differences between groups for CD4 T cells, CD4^+^/PD-1^+^ T cells, and B cells (Fig. 5B, C).

Finally, distinct populations of myeloid cells in tumor infiltrates were analyzed based on side scatter and the expression of CD11b and CD11c to distinguish CD11b^low^ (low scatter, monocyte-like), CD11b^high^ (high scatter, granulocyte-like), CD11c^+^/CD11b^−^ dendritic cells (conventional cDC1), and CD11c^+^/CD11b^+^ (other DC population). All were elevated in the Ig.Foldon-mLIGHT group compared to the membrane LIGHT group (and also compared to the mock control group, except for CD11c^+^/CD11b^+^ whose increase did not reach significance) (Fig. 5D).

The significant presence of various immune cell subpopulations, including myeloid cells and dendritic cells, indicates that LIGHT may not only enhance cytotoxic cell activity but also potentiate antigen presentation and overall immune activation within the tumor microenvironment.

## Discussion

This study describes a recombinant mouse LIGHT protein that binds to its receptors (mouse HVEM and LTβR), demonstrates activity in vitro through a cell-based reporter bioassay, and, when expressed by tumor cells, enhances anti-tumor activity in vivo in a poorly immunogenic cold tumor model by promoting immune cell infiltration. Biological assays are of crucial importance for the production and functional characterization of cytokine-like molecules such as native proteins and soluble recombinant forms of members of the TNF superfamily. The measurement of the biological activity of soluble recombinant human LIGHT has relied so far on the use of human tumor cell lines expressing either LTβR and/or HVEM [23, 33], or primary T cells expressing HVEM that can proliferate in response to LIGHT [34]. The cell-based bioassay developed in this study for mouse and human LIGHT is convenient, easy to quantify, and may discriminate between multimerization states and potencies of the ligands.

With the exception of mouse GITRL that was crystalized as a dimer [35], all other members of the TNF superfamily for which a structure is known, including human LIGHT, have a trimeric configuration and three receptor binding sites. Ligand-induced receptor clustering is at the apex of signaling in the TNF family [1]. Approaches commonly used to express active TNF family ligands (e.g., Flag-tagged or Ig-tagged) were unsuccessful for mouse LIGHT [2, 23], but insertion of a small homotrimeric domain in front of mouse LIGHT rescued receptor binding and bioactivity, probably by helping mLIGHT folding and/or stabilization in an active conformation.

Many patients do not respond to immune check-point blockade, especially those with low mutational load, meaning that complementary approaches are needed to overcome tumor resistance to this therapy. Targeting the LIGHT/HVEM/LTβR pathway is gaining attention in the immune-oncology field for several reasons [7, 36, 37]. Foundational studies on the biological function of LIGHT showed that the constitutive transgenic LIGHT expression under a T cell promoter leads to an autoimmune syndrome characterized by systemic tissue T cell infiltration and ensuing systemic inflammation [38]. Based on this observation, along with other reports claiming anti-tumor activity of membrane LIGHT expressed in immunogenic transplantable tumor cells lines [33, 39–43], we hypothesized that expression of a recombinant LIGHT could condition the tumor microenvironment and increase anti-tumor responses also in cold, non-immunogenic tumors. Data obtained with B16.F10 melanoma tumor cells expressing Ig.Foldon-mLIGHT support this hypothesis and additionally suggest that mLIGHT can act as a single agent to increase anti-tumor immunity. Previous work with transgenic expression of potent cytokines or chemokines such as GM-CSF, Flt3L, or IP-10 in B16 melanoma cells only displayed anti-tumor activity when combined with immune checkpoint blockade or after prophylactic immunization with irradiated cells expressing these cytokines prior to challenge with live tumor cells [30, 32], but never as single agents as observed with mLIGHT. The success of Ig.Foldon-mLIGHT but the failure of membrane LIGHT in the B16.F10 melanoma model helps to formulate further hypotheses regarding the mode of action of Ig.Foldon-mLIGHT. Immunogenicity generated by the transduction procedure is unlikely to be a decisive factor as membrane mLIGHT and Ig-Foldon-mLIGHT B16.F10 cells were comparable in that respect. Binding of mLIGHT to its receptors, in particular to HVEM, is insufficient for the observed anti-tumor effect because membrane LIGHT was active on mHVEM:Fas reporter cells but not on tumors. It is, however, difficult to compare concentrations and activities of soluble Ig.Foldon-mLIGHT versus membrane LIGHT that might be an intrinsically weaker activator of its cognate receptors. It is also tempting to hypothesize that soluble Ig.Foldon-mLIGHT must act at a distance of the tumor to exert activation and recruitment of immune cells, something that membrane LIGHT cannot do. This hypothesis is reinforced by the observation that membrane mLIGHT B16.F10 cells did not release any active soluble mLIGHT when cultured in vitro.

Ongoing cancer immunotherapy faces several challenges such as the need of promoting anti-tumor responses by bolstering and amplifying pre-existing anti-tumor responses against tumor-specific antigens or breaking tolerance to tumor-associated antigens. This therapeutic intervention by itself is insufficient to eliminate solid tumors, since tumor-specific T cells are known to be detectable in peripheral blood, but this is not sufficient since anti-tumor effector cells still need to traffic and infiltrate the tumor parenchyma [44, 45]. Once within the tumor, T cells must overcome the immunosuppressive conditions created by regulatory T cells (Tregs) and myeloid-derived suppressor cells for a successful outcome of immune checkpoint blockade [46]. The inability of effector cells to migrate into the tumor parenchyma in cold tumors may in part be explained by the observation that tumor vasculature differs from that of normal vasculature [47–51]. Despite the presence of inflammatory conditions, the vascular endothelium paradoxically lacks adhesion molecules (such as selectins and integrins), rendering it incapable of supporting the recruitment of immune cells trafficking through the tumor blood vessel network [49]. LIGHT appears to contribute favorably to counteract evasion mechanisms of tumors to subvert the anti-tumor response, thereby enhancing the ability of immune cells to infiltrate the tumor and exert anti-tumor effects [50, 52].

The recombinant expression of mLIGHT offers clear advantages over other immunostimulatory molecules as it can influence both immune cell infiltrates and stromal cells of the TME. On the one hand, the LT/LIGHT/HVEM (LTα3/HVEM and LIGHT/HVEM) bidirectional interaction can drive myeloid cell activation, licensing of dendritic cells for antigen presentation, NK cell activation, and T cell co-stimulation, thus promoting anti-tumor responses [4, 37, 53, 54]. The engagement of HVEM by LIGHT co-stimulates T cells in a CD28-independent manner [14, 19]. The engagement of LTβR by LIGHT on DCs seems to compensate for the absence of CD40 ligation in the licensing of DC, a signal required for appropriate T cell co-stimulation [20, 21]. Moreover, lymphotoxin and LIGHT can engage LTβR on stromal components of TME, such as fibroblasts and endothelial cells promoting the secretion of chemokines and contributing to endothelial cell activation, accompanied by increased expression of adhesion molecules, resulting at the end in the recruitment of effector cells and the subsequent control of tumor growth [37, 50, 55]. The recruitment of both populations of cytotoxic cells, innate NK cells and adaptive CD8 T cells, is required for an effective anti-tumor response. Indeed, NK cells alone do not reject tumors in Rag-deficient mice and NK cell depletion in WT mice prevents effective CTL responses [44, 56]. This close link between NK cells, T cells, and DCs and effective clinical anti-tumor responses has been detected intratumorally in patients under the cover of immune checkpoint blockade [57, 58].

Despite the moderate in vitro killing activity of Ig.Foldon-mouse LIGHT and the fact that purification affected to some extent its functional activity (requiring higher concentrations to achieve a similar effect in the cell reporter bioassay), the proper folding facilitated by Foldon restored the function lost in Ig.mouse LIGHT. As a result, the EC-50 for Ig.Foldon-LIGHT transduced tumor cells was 1.9 ng/ml, showing better performance than the purified form (EC50 of 51 ng/ml) of the same protein in the cell reporter bioassay.

Our findings indicate that recombinant mouse LIGHT (not human LIGHT) in a preclinical mouse model can promote beneficial responses through the recruitment of critical immune effector cells (cytotoxic cells and dendritic cells) in the tumor parenchyma.

Future studies should address whether combining immune checkpoint blockade and soluble Ig.Foldon-mLIGHT in the B16.F10 model are synergistic approaches capable of eradicating melanoma tumors and induce systemic immunity. Another aspect worth assessing in terms of potential translation to humans is to unveil whether human Ig-hLIGHT (that unlike Ig.mLIGHT is active on its own) can induce comparable anti-tumor responses. This would be a relevant experimental approach since human LIGHT can bind mouse LTβR and mouse HVEM. Furthermore, LIGHT-transduced T cells could enhance current adoptive CAR (chimeric antigen receptor) T cell therapy by activating stromal cells and endothelial cells to favor the transmigration and invasion of bystander T cells.

## Supplementary Information

Below is the link to the electronic supplementary material.

ESM 1 (PNG 90.7 KB)

Supplementary file1 (TIF 91 KB)

ESM 2 (PNG 103.7 KB)

Supplementary file2 (TIF 103 KB)

ESM 3 (PNG 123 KB)

Supplementary file3 (TIF 124 KB)

ESM 4 (PNG 217 KB)

Supplementary file4 (TIF 217 KB)

ESM 5 (PNG 289 KB)

Supplementary file5 (TIF 290 KB)

ESM 6 (PNG 939 KB)

Supplementary file6 (TIF 939 KB)

## Data Availability

The data generated in this study are available upon request from the corresponding author.

## Abbreviations

LIGHT: Homologous to lymphotoxin, exhibits inducible expression and competes with HSV glycoprotein D for binding to herpesvirus entry mediator, a receptor expressed on T lymphocytes
mAb: Monoclonal antibody
CD: Cluster of differentiation
HSV: Herpesvirus
TNF: Tumor necrosis factor
TNFR: Tumor necrosis factor receptor
HVEM: Herpesvirus entry mediator
LTβR: Lymphotoxin β receptor
CRD: Cysteine-rich domain
APC: Antigen-presenting cells
CTL: Cytotoxic T lymphocyte
eGFP: Enhanced green fluorescent protein
APC: Allophycocyanin
PE: Phycoerythrin
MHC: Major histocompatibility complex
Ig-LIGHT: Immunoglobulin IgG2a Fc-tagged mouse LIGHT
Ig.Foldon-mLIGHT: Immunoglobulin IgG2a Fc (Hinge-CH2-CH2).Foldon-tagged soluble mouse LIGHT
NK: Natural killer
NKT: Natural killer T cells
TCR: T cell receptor
PI: Propidium iodide
FCS: Fetal calf serum
SD: Standard deviation
PBS: Phosphate buffer saline
BTLA: B-and T-lymphocyte attenuator
LTα: Lymphotoxin alpha
PD-1: Programmed cell death – 1
ECD: Extracellular domain
TNFSF: Tumor necrosis factor superfamily
TNFRSF: Tumor necrosis factor receptor superfamily
CRD: Cysteine-rich domains
THD: TNF homology domain
TME: Tumor microenvironment
TILs: Tumor-infiltrating lymphocytes
TM: Transmembrane
IC: Intracellular
SA: Streptavidin
AF: Alexa fluor
FSC: Forward scatter
SSC: Side scatter

## References

[1] Bodmer JL, Schneider P, Tschopp J (2002) The molecular architecture of the TNF superfamily. Trends BiochemSci 27:19–26 10.1016/s0968-0004(01)01995-8 11796220

[2] Bossen C, Ingold K, Tardivel A, Bodmer JL, Gaide O, Hertig S, Ambrose C, Tschopp J, Schneider P (2006) Interactions of tumor necrosis factor (TNF) and TNF receptor family members in the mouse and human. JBiolChem 281:13964–13971 10.1074/jbc.M601553200 16547002

[3] Granger SW, Ware CF (2001) Turning on LIGHT JClinInvest 108:1741–1742 10.1172/JCI14651 PMC209475 11748255

[4] Yu P, Fu YX (2008) Targeting tumors with LIGHT to generate metastasis-clearing immunity. Cytokine Growth Factor Rev 19:285–294. 10.1016/j.cytogfr.2008.04.004 18508404 10.1016/j.cytogfr.2008.04.004 PMC2517180

[5] Mauri DN, Ebner R, Montgomery RI, Kochel KD, Cheung TC, Yu GL, Ruben S, Murphy M, Eisenberg RJ, Cohen GH et al (1998) LIGHT, a new member of the TNF superfamily, and lymphotoxin alpha are ligands for herpesvirus entry mediator. Immunity 8:21–30 9462508 10.1016/s1074-7613(00)80455-0

[6] Del Rio ML, Fernandez-Renedo C, Chaloin O, Scheu S, Pfeffer K, Shintani Y, Perez-Simon JA, Schneider P, Rodriguez-Barbosa JI (2016) Immunotherapeutic targeting of LIGHT/LTbetaR/HVEM pathway fully recapitulates the reduced cytotoxic phenotype of LIGHT-deficient T cells. mAbs: 1–13 10.1080/19420862.2015.1132130 PMC4966841 26752542

[7] Ware CF, Croft M, Neil GA (2022) Realigning the LIGHT signaling network to control dysregulated inflammation. The Journal of experimental medicine 219. 10.1084/jem.20220236 10.1084/jem.20220236 PMC9130030 35604387

[8] Del Rio ML, Jones ND, Buhler L, Norris P, Shintani Y, Ware CF, Rodriguez-Barbosa JI (2012) Selective blockade of herpesvirus entry mediator-B and T lymphocyte attenuator pathway ameliorates acute graft-versus-host reaction. JImmunol 188:4885–4896 22490863 10.4049/jimmunol.1103698 PMC3925259

[9] Seo GY, Takahashi D, Wang Q, Mikulski Z, Chen A, Chou TF, Marcovecchio P, McArdle S, Sethi A, Shui JW et al (2022) Epithelial HVEM maintains intraepithelial T cell survival and contributes to host protection. Science immunology 7:eabm6931. 10.1126/sciimmunol.abm6931 35905286 10.1126/sciimmunol.abm6931 PMC9422995

[10] Androlewicz MJ, Browning JL, Ware CF (1992) Lymphotoxin is expressed as a heteromeric complex with a distinct 33-kDa glycoprotein on the surface of an activated human T cell hybridoma. JBiolChem 267:2542–2547 1733951

[11] Giles DA, Zahner S, Krause P, Van Der Gracht E, Riffelmacher T, Morris V, Tumanov A, Kronenberg M (2018) The tumor necrosis factor superfamily members TNFSF14 (LIGHT), lymphotoxin beta and lymphotoxin beta receptor interact to regulate intestinal inflammation. Front Immunol 9:2585. 10.3389/fimmu.2018.02585 30524422 10.3389/fimmu.2018.02585 PMC6262400

[12] Rennert PD, James D, Mackay F, Browning JL, Hochman PS (1998) Lymph node genesis is induced by signaling through the lymphotoxin beta receptor. Immunity 9:71–79 9697837 10.1016/s1074-7613(00)80589-0

[13] van de Pavert SA, Mebius RE (2010) New insights into the development of lymphoid tissues. Nat Rev Immunol 10:664–674. 10.1038/nri2832 20706277 10.1038/nri2832

[14] Shi G, Luo H, Wan X, Salcedo TW, Zhang J, Wu J (2002) Mouse T cells receive costimulatory signals from LIGHT, a TNF family member. Blood 100:3279–3286 12384428 10.1182/blood-2002-05-1404

[15] Murphy TL, Murphy KM (2010) Slow down and survive: enigmatic immunoregulation by BTLA and HVEM. AnnuRevImmunol 28:389–411 10.1146/annurev-immunol-030409-101202 20307212

[16] Scheu S, Alferink J, Potzel T, Barchet W, Kalinke U, Pfeffer K (2002) Targeted disruption of LIGHT causes defects in costimulatory T cell activation and reveals cooperation with lymphotoxin beta in mesenteric lymph node genesis. JExpMed 195:1613–1624 10.1084/jem.20020215 PMC2193565 12070288

[17] Del Rio ML, Schneider P, Fernandez-Renedo C, Perez-Simon JA, Rodriguez-Barbosa JI (2013) LIGHT/HVEM/LTbetaR interaction as a target for the modulation of the allogeneic immune response in transplantation. AmJTransplant 13:541–551 10.1111/ajt.12089 23356438

[18] Yoo KJ, Johannes K, González LE, Patel A, Shuptrine CW, Opheim Z, Lenz K, Campbell K, Nguyen TA, Miriyala J, et al. (2022) LIGHT (TNFSF14) costimulation enhances myeloid cell activation and antitumor immunity in the setting of PD-1/PD-L1 and TIGIT checkpoint blockade. Journal of immunology (Baltimore, Md : 1950). 10.4049/jimmunol.2101175 10.4049/jimmunol.2101175 PMC10580117 35817517

[19] Steinberg MW, Cheung TC, Ware CF (2011) The signaling networks of the herpesvirus entry mediator (TNFRSF14) in immune regulation. ImmunolRev 244:169–187 10.1111/j.1600-065X.2011.01064.x PMC3381650 22017438

[20] Holmes TD, Wilson EB, Black EV, Benest AV, Vaz C, Tan B, Tanavde VM, Cook GP (2014) Licensed human natural killer cells aid dendritic cell maturation via TNFSF14/LIGHT. Proc Natl Acad Sci USA 111:E5688-5696. 10.1073/pnas.1411072112 25512551 10.1073/pnas.1411072112 PMC4284554

[21] Morel Y, Truneh A, Sweet RW, Olive D, Costello RT (2001) The TNF superfamily members LIGHT and CD154 (CD40 ligand) costimulate induction of dendritic cell maturation and elicit specific CTL activity. JImmunol 167:2479–2486 11509586 10.4049/jimmunol.167.5.2479

[22] Wang Y, Zhu M, Miller M, Fu YX (2009) Immunoregulation by tumor necrosis factor superfamily member LIGHT. ImmunolRev 229:232–243 10.1111/j.1600-065X.2009.00762.x PMC4634935 19426225

[23] Ito T, Iwamoto K, Tsuji I, Tsubouchi H, Omae H, Sato T, Ohba H, Kurokawa T, Taniyama Y, Shintani Y (2011) Trimerization of murine TNF ligand family member LIGHT increases the cytotoxic activity against the FM3A mammary carcinoma cell line. ApplMicrobiolBiotechnol 90:1691–1699 10.1007/s00253-011-3168-8 21400099

[24] Schneider P, Willen L, Smulski CR (2014) Tools and techniques to study ligand-receptor interactions and receptor activation by TNF superfamily members. Methods Enzymol 545:103–125. 10.1016/b978-0-12-801430-1.00005-6 25065888 10.1016/B978-0-12-801430-1.00005-6

[25] Gao X, Huang L (1995) Cationic liposome-mediated gene transfer. Gene Ther 2:710–722 8750010

[26] Young L, Sung J, Stacey G, Masters JR (2010) Detection of mycoplasma in cell cultures. Nat Protoc 5:929–934. 10.1038/nprot.2010.43 20431538 10.1038/nprot.2010.43

[27] Fidler IJ, Kripke ML (1977) Metastasis results from preexisting variant cells within a malignant tumor. Science (New York, NY) 197:893–895. 10.1126/science.887927 10.1126/science.887927 887927

[28] Allard B, Allard D, Stagg J (2016) Methods to evaluate the antitumor activity of immune checkpoint inhibitors in preclinical studies. Methods in molecular biology (Clifton, NJ) 1458:159–177. 10.1007/978-1-4939-3801-8_12 10.1007/978-1-4939-3801-8_12 27581021

[29] Schneider P, Holler N, Bodmer JL, Hahne M, Frei K, Fontana A, Tschopp J (1998) Conversion of membrane-bound Fas(CD95) ligand to its soluble form is associated with downregulation of its proapoptotic activity and loss of liver toxicity. J Exp Med 187:1205–1213. 10.1084/jem.187.8.1205 9547332 10.1084/jem.187.8.1205 PMC2212219

[30] Dranoff G, Jaffee E, Lazenby A, Golumbek P, Levitsky H, Brose K, Jackson V, Hamada H, Pardoll D, Mulligan RC (1993) Vaccination with irradiated tumor cells engineered to secrete murine granulocyte-macrophage colony-stimulating factor stimulates potent, specific, and long-lasting anti-tumor immunity. Proc Natl Acad Sci USA 90:3539–3543 8097319 10.1073/pnas.90.8.3539 PMC46336

[31] Curran MA, Allison JP (2009) Tumor vaccines expressing flt3 ligand synergize with ctla-4 blockade to reject preimplanted tumors. Can Res 69:7747–7755. 10.1158/0008-5472.Can-08-3289 10.1158/0008-5472.CAN-08-3289 PMC2756314 19738077

[32] van Elsas A, Hurwitz AA, Allison JP (1999) Combination immunotherapy of B16 melanoma using anti-cytotoxic T lymphocyte-associated antigen 4 (CTLA-4) and granulocyte/macrophage colony-stimulating factor (GM-CSF)-producing vaccines induces rejection of subcutaneous and metastatic tumors accompanied by autoimmune depigmentation. J Exp Med 190:355–366 10430624 10.1084/jem.190.3.355 PMC2195583

[33] Zhai Y, Guo R, Hsu TL, Yu GL, Ni J, Kwon BS, Jiang GW, Lu J, Tan J, Ugustus M et al (1998) LIGHT, a novel ligand for lymphotoxin beta receptor and TR2/HVEM induces apoptosis and suppresses in vivo tumor formation via gene transfer. JClinInvest 102:1142–1151 10.1172/JCI3492 PMC509097 9739048

[34] Harrop JA, McDonnell PC, Brigham-Burke M, Lyn SD, Minton J, Tan KB, Dede K, Spampanato J, Silverman C, Hensley P et al (1998) Herpesvirus entry mediator ligand (HVEM-L), a novel ligand for HVEM/TR2, stimulates proliferation of T cells and inhibits HT29 cell growth. JBiolChem 273:27548–27556 10.1074/jbc.273.42.27548 9765287

[35] Zhou Z, Tone Y, Song X, Furuuchi K, Lear JD, Waldmann H, Tone M, Greene MI, Murali R (2008) Structural basis for ligand-mediated mouse GITR activation. Proc Natl Acad Sci USA 105:641–645. 10.1073/pnas.0711206105 18178614 10.1073/pnas.0711206105 PMC2206589

[36] Shuptrine CW, Perez VM, Selitsky SR, Schreiber TH, Fromm G (2023) Shining a LIGHT on myeloid cell targeted immunotherapy. European journal of cancer (Oxford, England, (1990) 187:147–160. 10.1016/j.ejca.2023.03.040 37167762 10.1016/j.ejca.2023.03.040

[37] Skeate JG, Otsmaa ME, Prins R, Fernandez DJ, Da Silva DM, Kast WM (2020) TNFSF14: LIGHTing the way for effective cancer immunotherapy. Front Immunol 11:922. 10.3389/fimmu.2020.00922 32499782 10.3389/fimmu.2020.00922 PMC7243824

[38] Shaikh RB, Santee S, Granger SW, Butrovich K, Cheung T, Kronenberg M, Cheroutre H, Ware CF (2001) Constitutive expression of LIGHT on T cells leads to lymphocyte activation, inflammation, and tissue destruction. JImmunol 167:6330–6337 11714797 10.4049/jimmunol.167.11.6330

[39] Yu P, Lee Y, Liu W, Chin RK, Wang J, Wang Y, Schietinger A, Philip M, Schreiber H, Fu YX (2004) Priming of naive T cells inside tumors leads to eradication of established tumors. Nat Immunol 5:141–149. 10.1038/ni1029 14704792 10.1038/ni1029

[40] Tamada K, Shimozaki K, Chapoval AI, Zhu G, Sica G, Flies D, Boone T, Hsu H, Fu YX, Nagata S et al (2000) Modulation of T-cell-mediated immunity in tumor and graft-versus-host disease models through the LIGHT co-stimulatory pathway. NatMed 6:283–289 10.1038/73136 10700230

[41] Yu P, Lee Y, Wang Y, Liu X, Auh S, Gajewski TF, Schreiber H, You Z, Kaynor C, Wang X et al (2007) Targeting the primary tumor to generate CTL for the effective eradication of spontaneous metastases. Journal of immunology (Baltimore, Md, et al (1950) 179:1960–1968. 10.4049/jimmunol.179.3.1960 10.4049/jimmunol.179.3.1960 PMC2387226 17641063

[42] Hisada M, Yoshimoto T, Kamiya S, Magami Y, Miyaji H, Yoneto T, Tamada K, Aoki T, Koyanagi Y, Mizuguchi J (2004) Synergistic antitumor effect by coexpression of chemokine CCL21/SLC and costimulatory molecule LIGHT. Cancer Gene Ther 11:280–288. 10.1038/sj.cgt.7700676 15002032 10.1038/sj.cgt.7700676

[43] Hu G, Liu Y, Li H, Zhao D, Yang L, Shen J, Hong X, Cao X, Wang Q (2010) Adenovirus-mediated LIGHT gene modification in murine B-cell lymphoma elicits a potent antitumor effect. Cell Mol Immunol 7:296–305. 10.1038/cmi.2010.15 20418899 10.1038/cmi.2010.15 PMC4003227

[44] Melero I, Rouzaut A, Motz GT, Coukos G (2014) T-cell and NK-cell infiltration into solid tumors: a key limiting factor for efficacious cancer immunotherapy. Cancer Discov 4:522–526. 10.1158/2159-8290.cd-13-0985 24795012 10.1158/2159-8290.CD-13-0985 PMC4142435

[45] Zhang J, Huang D, Saw PE, Song E (2022) Turning cold tumors hot: from molecular mechanisms to clinical applications. Trends in immunology. 10.1016/j.it.2022.04.010 10.1016/j.it.2022.04.010 35624021

[46] Veglia F, Sanseviero E, Gabrilovich DI (2021) Myeloid-derived suppressor cells in the era of increasing myeloid cell diversity. Nature reviews Immunology: 1–14. 10.1038/s41577-020-00490-y 10.1038/s41577-020-00490-y PMC7849958 33526920

[47] Ganss R, Arnold B, Hämmerling GJ (2004) Mini-review: overcoming tumor-intrinsic resistance to immune effector function. Eur J Immunol 34:2635–2641. 10.1002/eji.200425474 15368278 10.1002/eji.200425474

[48] Quezada SA, Peggs KS, Simpson TR, Shen Y, Littman DR, Allison JP (2008) Limited tumor infiltration by activated T effector cells restricts the therapeutic activity of regulatory T cell depletion against established melanoma. J Exp Med 205:2125–2138. 10.1084/jem.20080099 18725522 10.1084/jem.20080099 PMC2526206

[49] Yang T, Xiao H, Liu X, Wang Z, Zhang Q, Wei N, Guo X (2021) Vascular normalization: a new window opened for cancer therapies. Front Oncol 11:719836. 10.3389/fonc.2021.719836 34476218 10.3389/fonc.2021.719836 PMC8406857

[50] He B, Jabouille A, Steri V, Johansson-Percival A, Michael IP, Kotamraju VR, Junckerstorff R, Nowak AK, Hamzah J, Lee G et al (2018) Vascular targeting of LIGHT normalizes blood vessels in primary brain cancer and induces intratumoural high endothelial venules. J Pathol 245:209–221. 10.1002/path.5080 29603739 10.1002/path.5080 PMC6737176

[51] Johansson-Percival A, He B, Li ZJ, Kjellén A, Russell K, Li J, Larma I, Ganss R (2017) De novo induction of intratumoral lymphoid structures and vessel normalization enhances immunotherapy in resistant tumors. Nat Immunol 18:1207–1217. 10.1038/ni.3836 28892469 10.1038/ni.3836

[52] He B, Johansson-Percival A, Backhouse J, Li J, Lee GYF, Hamzah J, Ganss R (2020) Remodeling of metastatic vasculature reduces lung colonization and sensitizes overt metastases to immunotherapy. Cell Rep 30(714–724):e715. 10.1016/j.celrep.2019.12.013 10.1016/j.celrep.2019.12.013 31968248

[53] Fan Z, Yu P, Wang Y, Wang Y, Fu ML, Liu W, Sun Y, Fu YX (2006) NK-cell activation by LIGHT triggers tumor-specific CD8+ T-cell immunity to reject established tumors. Blood 107:1342–1351 16223768 10.1182/blood-2005-08-3485 PMC1895398

[54] Stringhini M, Mock J, Fontana V, Murer P, Neri D (2021) Antibody-mediated delivery of LIGHT to the tumor boosts natural killer cells and delays tumor progression. mAbs 13: 1868066. 10.1080/19420862.2020.1868066 10.1080/19420862.2020.1868066 PMC7808322 33404287

[55] Johansson-Percival A, Ganss R (2021) Therapeutic induction of tertiary lymphoid structures in cancer through stromal remodeling. Front Immunol 12:674375. 10.3389/fimmu.2021.674375 34122434 10.3389/fimmu.2021.674375 PMC8191417

[56] Kyrysyuk O, Wucherpfennig KW (2022) Designing cancer immunotherapies that engage T cells and NK cells. Annual review of immunology 41. 10.1146/annurev-immunol-101921-044122 10.1146/annurev-immunol-101921-044122 PMC10159905 36446137

[57] Barry KC, Hsu J, Broz ML, Cueto FJ, Binnewies M, Combes AJ, Nelson AE, Loo K, Kumar R, Rosenblum MD et al (2018) A natural killer-dendritic cell axis defines checkpoint therapy-responsive tumor microenvironments. Nat Med 24:1178–1191. 10.1038/s41591-018-0085-8 29942093 10.1038/s41591-018-0085-8 PMC6475503

[58] Martinet L, Garrido I, Filleron T, Le Guellec S, Bellard E, Fournie JJ, Rochaix P, Girard JP (2011) Human solid tumors contain high endothelial venules: association with T- and B-lymphocyte infiltration and favorable prognosis in breast cancer. Can Res 71:5678–5687. 10.1158/0008-5472.Can-11-0431 10.1158/0008-5472.CAN-11-0431 21846823

[59] Del Rio ML, Kaye J, Rodriguez-Barbosa JI (2010) Detection of protein on BTLA(low) cells and in vivo antibody-mediated down-modulation of BTLA on lymphoid and myeloid cells of C57BL/6 and BALB/c BTLA allelic variants. Immunobiology 215:570–578 19837478 10.1016/j.imbio.2009.09.008

[60] Unkeless JC (1979) Characterization of a monoclonal antibody directed against mouse macrophage and lymphocyte Fc receptors. JExpMed 150:580–596 10.1084/jem.150.3.580 PMC2185638 90108

