# Supplementary figures and images for: A bioactive soluble recombinant mouse LIGHT promotes effective tumor immune cell infiltration delaying tumor growth
Source: J Mol Med (Berl). 2025 Jun 2;103(7):867–83. doi: 10.1007/s00109-025-02552-x (PMC12287161; doi:10.1007/s00109-025-02552-x)

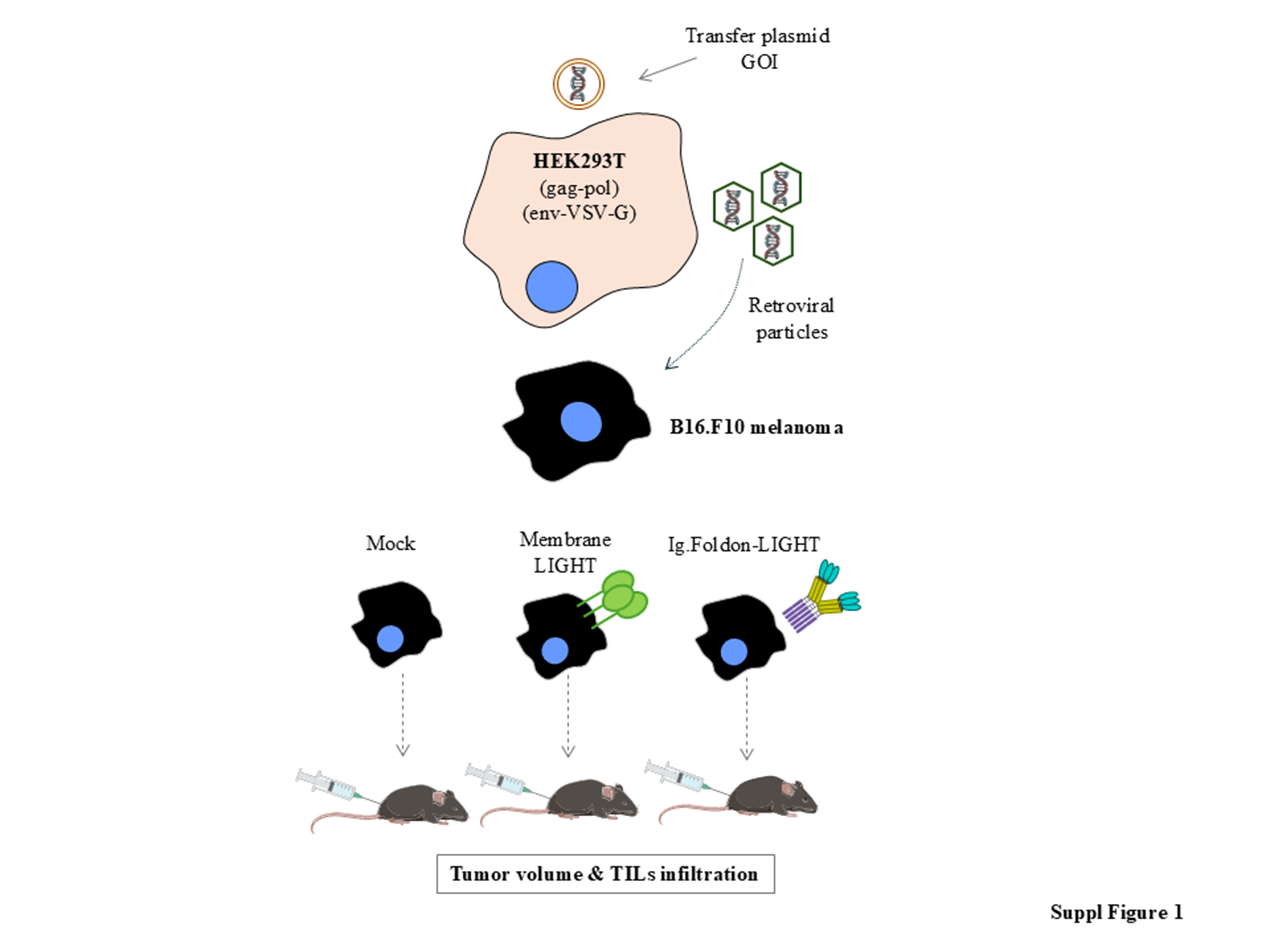

Supplement: Supplementary file 1 — (PNG 90.7 KB) [file 109_2025_2552_Fig6_ESM.png]

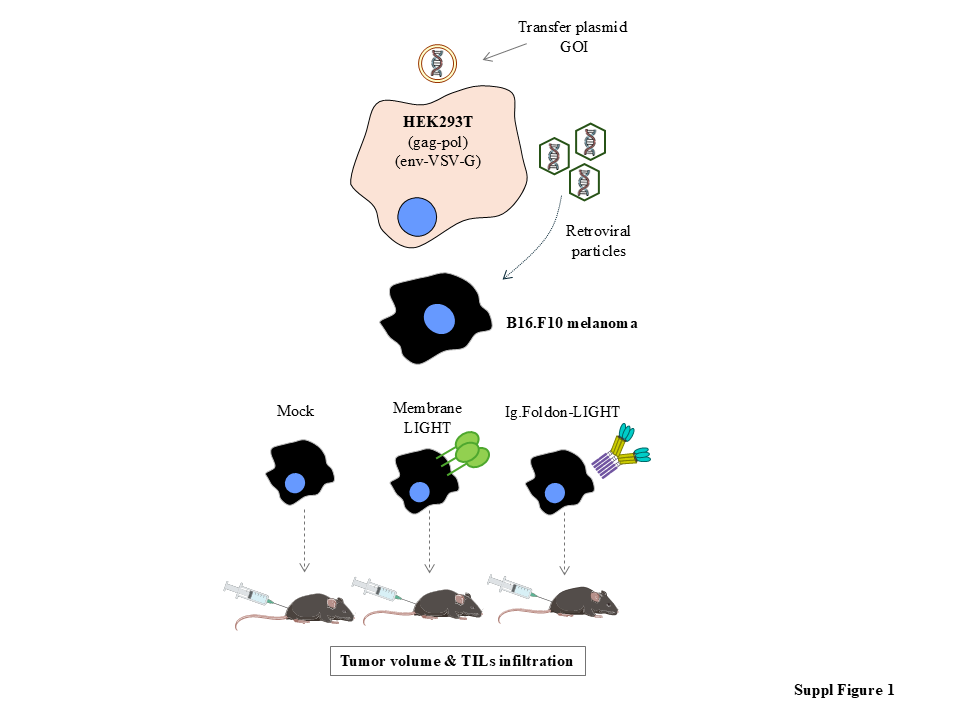

Supplement: Supplementary file 2 — Supplementary file1 (TIF 91 KB) [file 109_2025_2552_MOESM1_ESM.tif]

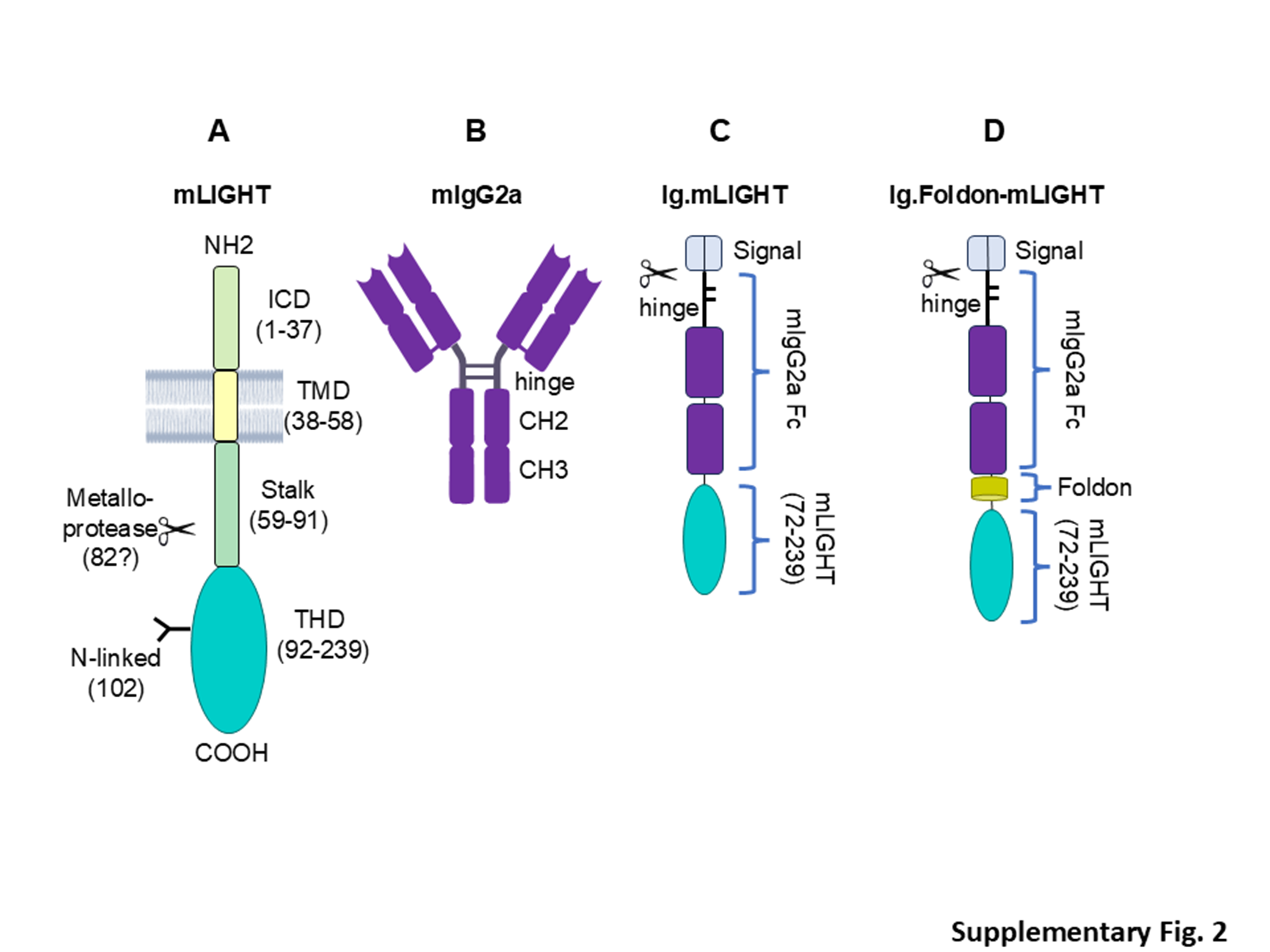

Supplement: Supplementary file 3 — (PNG 103.7 KB) [file 109_2025_2552_Fig7_ESM.png]

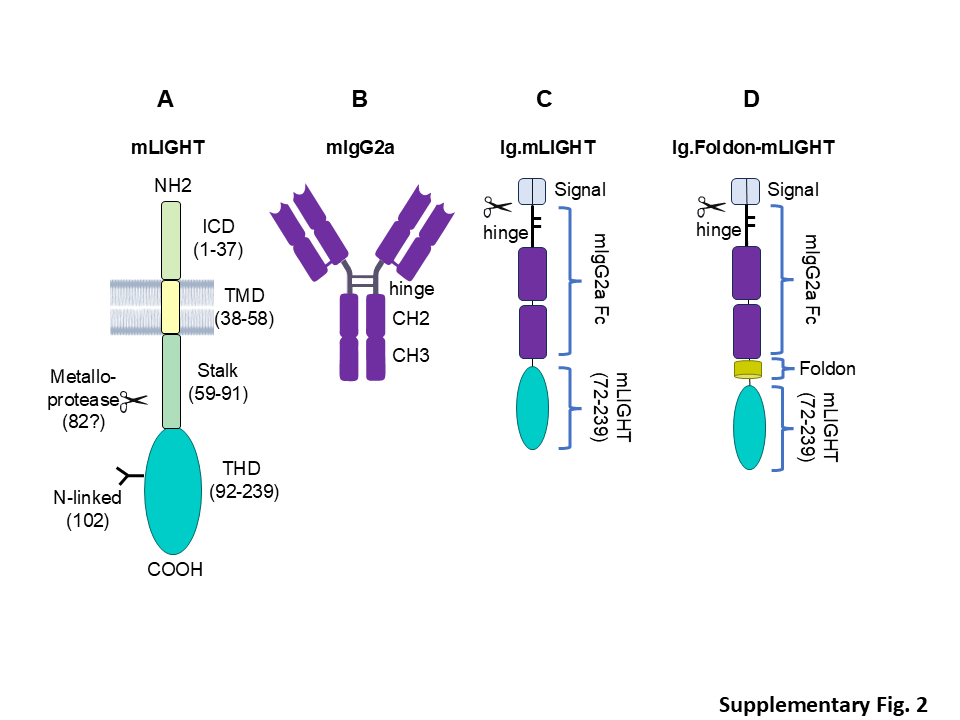

Supplement: Supplementary file 4 — Supplementary file2 (TIF 103 KB) [file 109_2025_2552_MOESM2_ESM.tif]

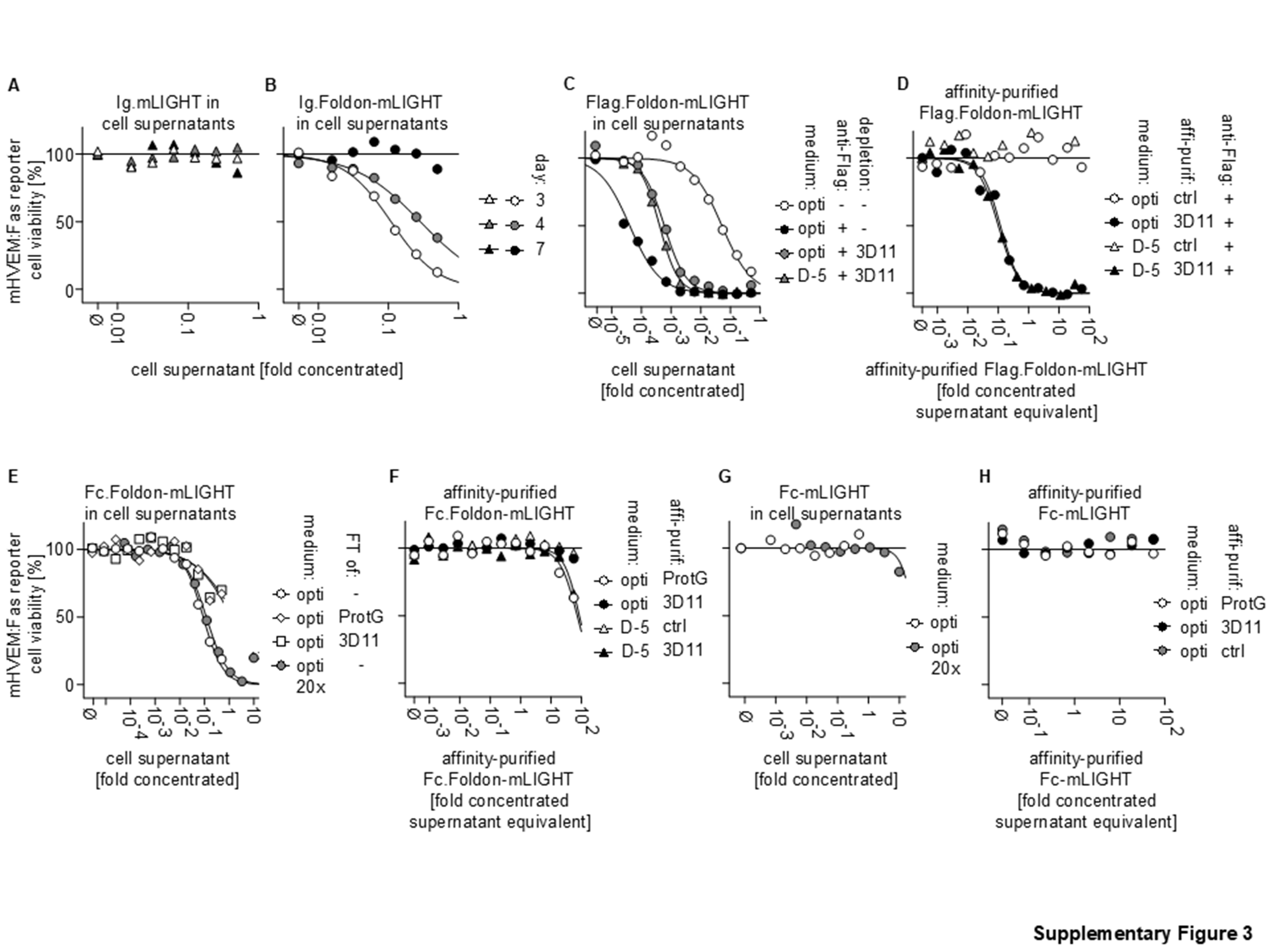

Supplement: Supplementary file 5 — (PNG 123 KB) [file 109_2025_2552_Fig8_ESM.png]

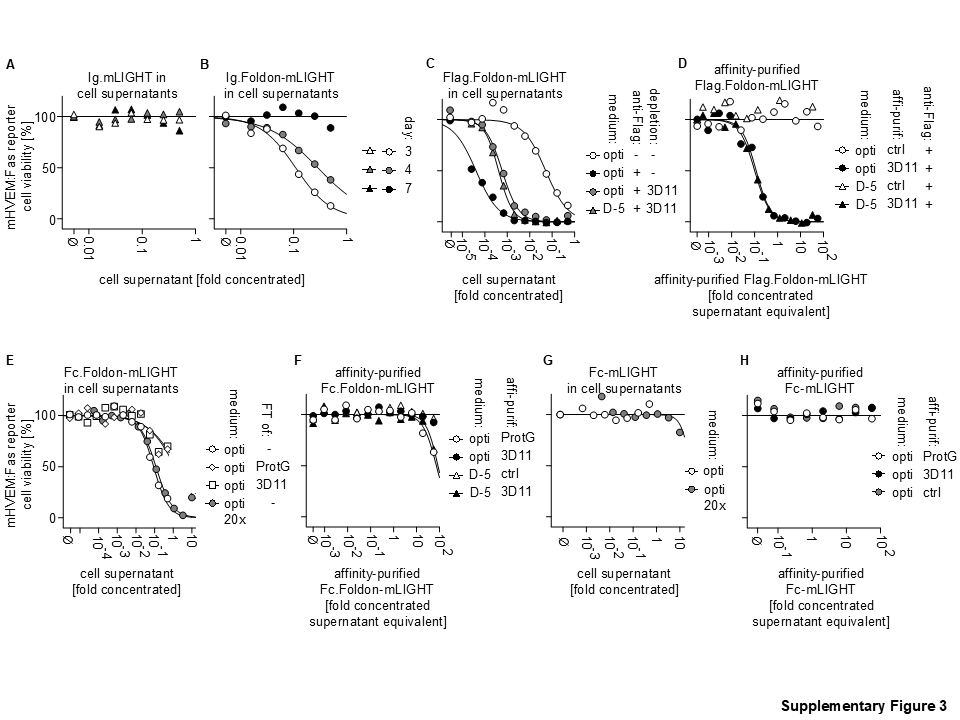

Supplement: Supplementary file 6 — Supplementary file3 (TIF 124 KB) [file 109_2025_2552_MOESM3_ESM.tif]

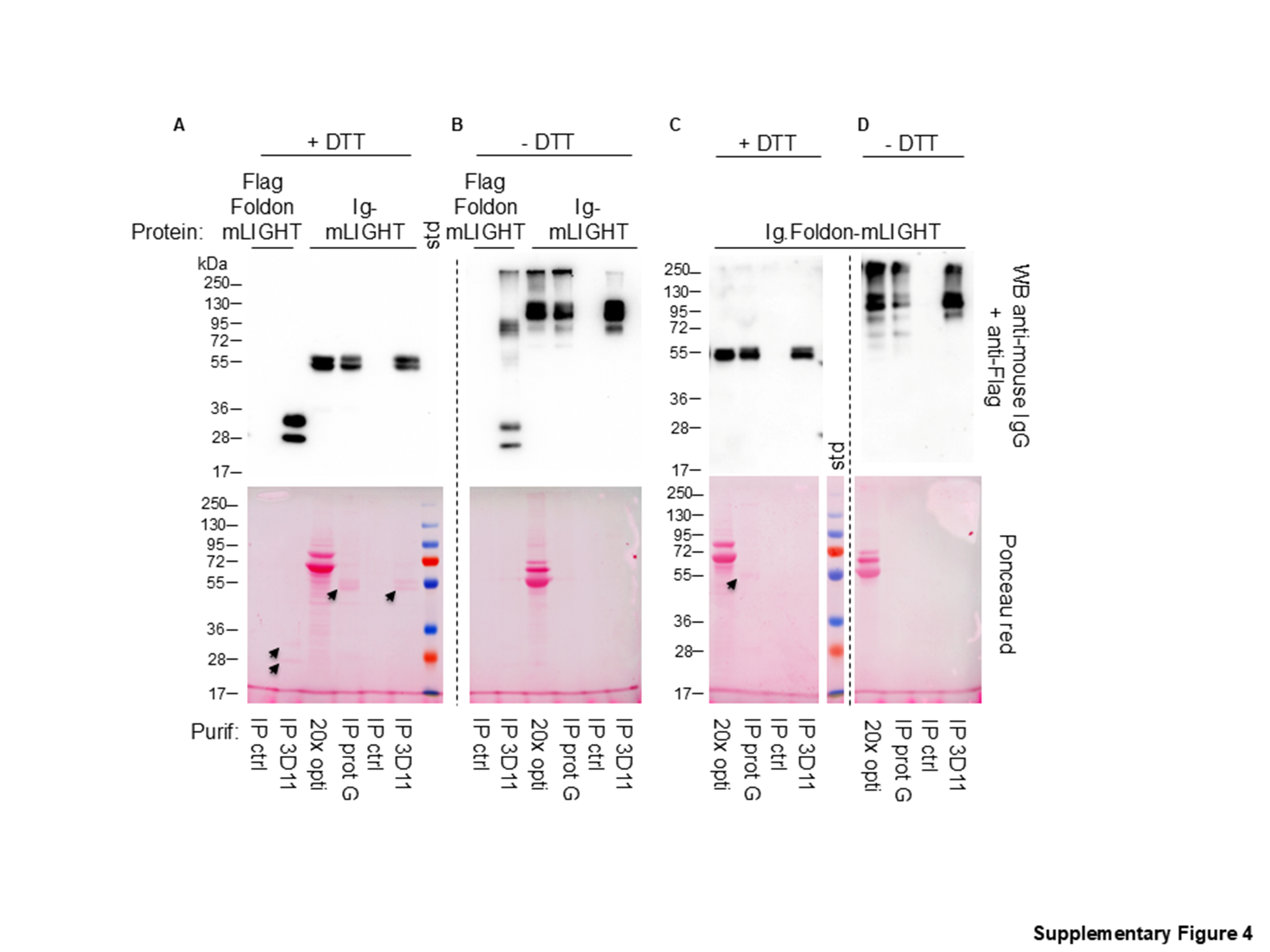

Supplement: Supplementary file 7 — (PNG 217 KB) [file 109_2025_2552_Fig9_ESM.png]

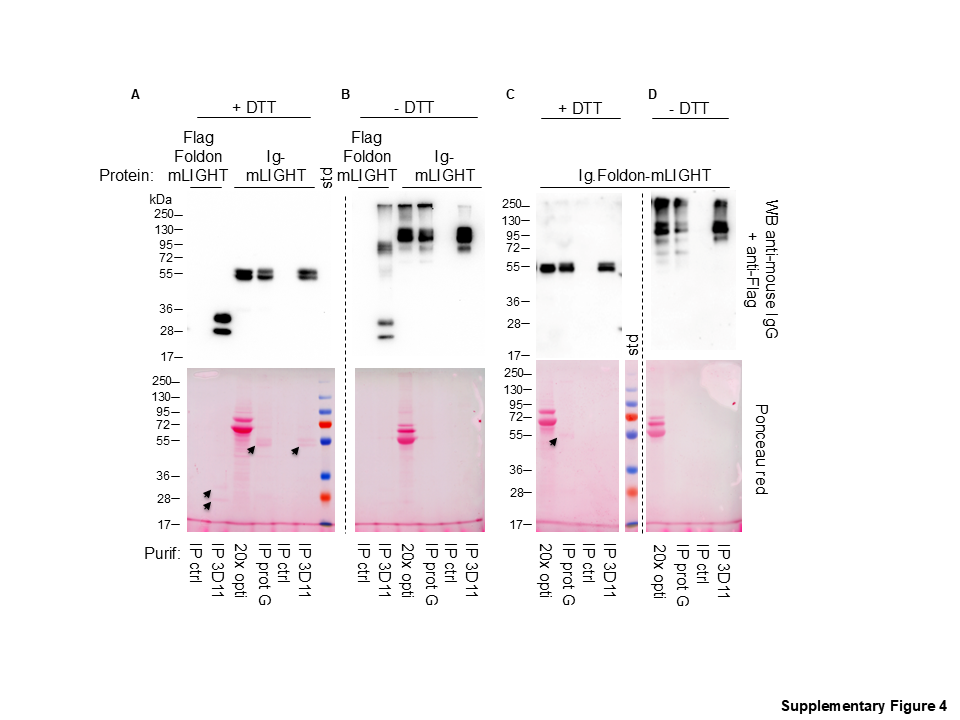

Supplement: Supplementary file 8 — Supplementary file4 (TIF 217 KB) [file 109_2025_2552_MOESM4_ESM.tif]

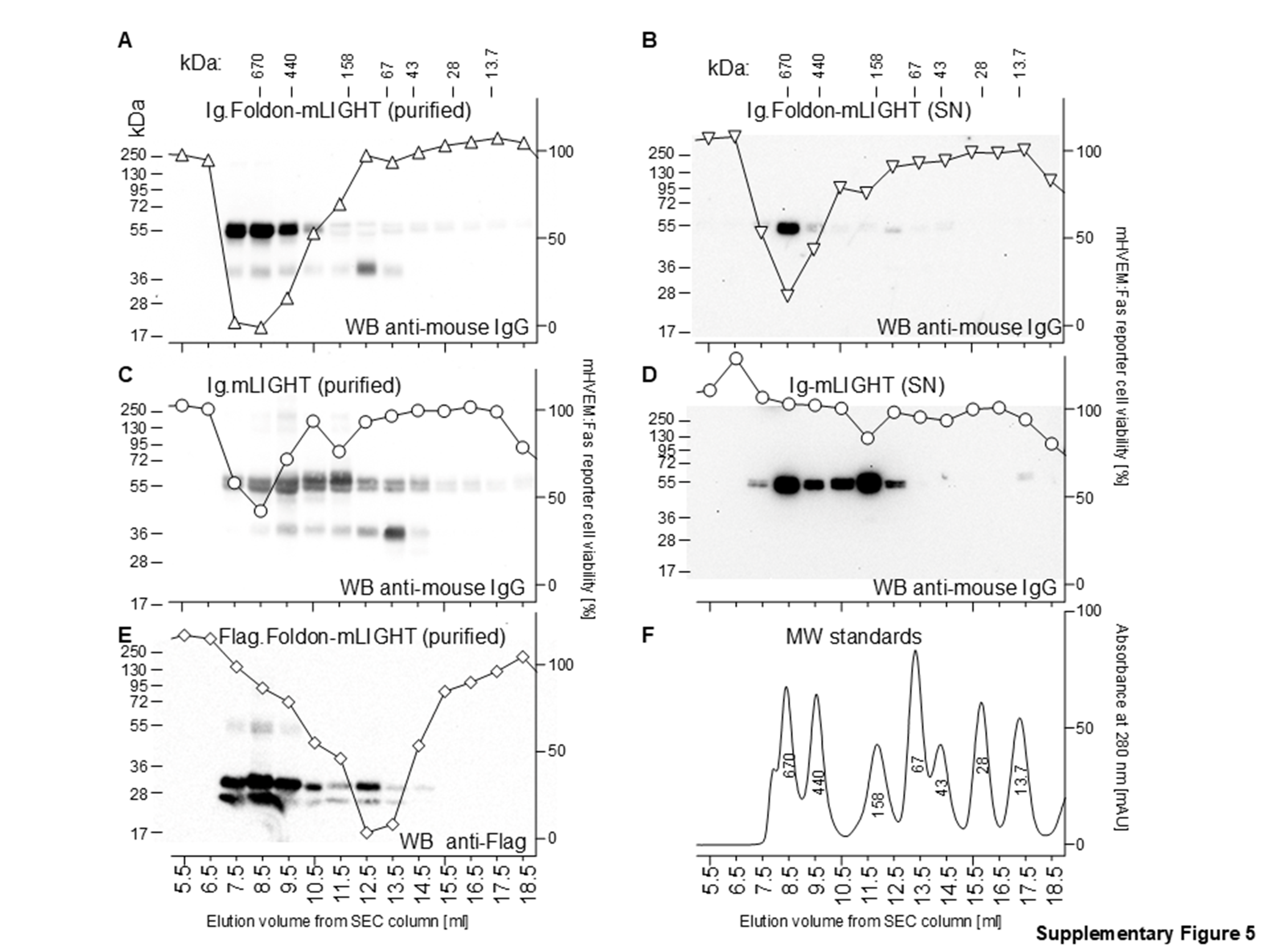

Supplement: Supplementary file 9 — (PNG 289 KB) [file 109_2025_2552_Fig10_ESM.png]

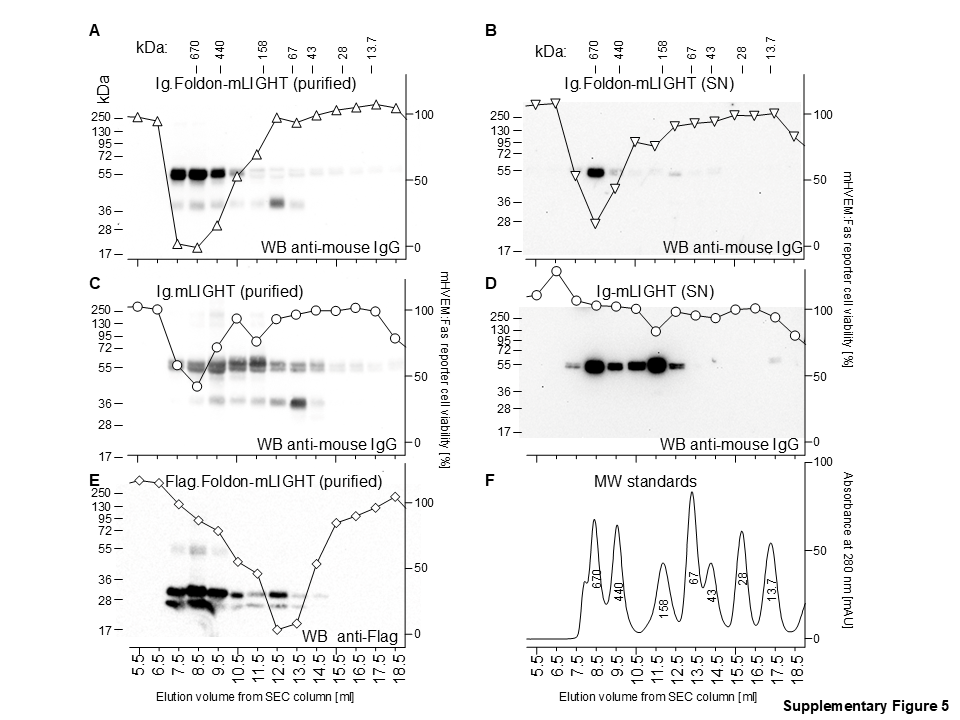

Supplement: Supplementary file 10 — Supplementary file5 (TIF 290 KB) [file 109_2025_2552_MOESM5_ESM.tif]

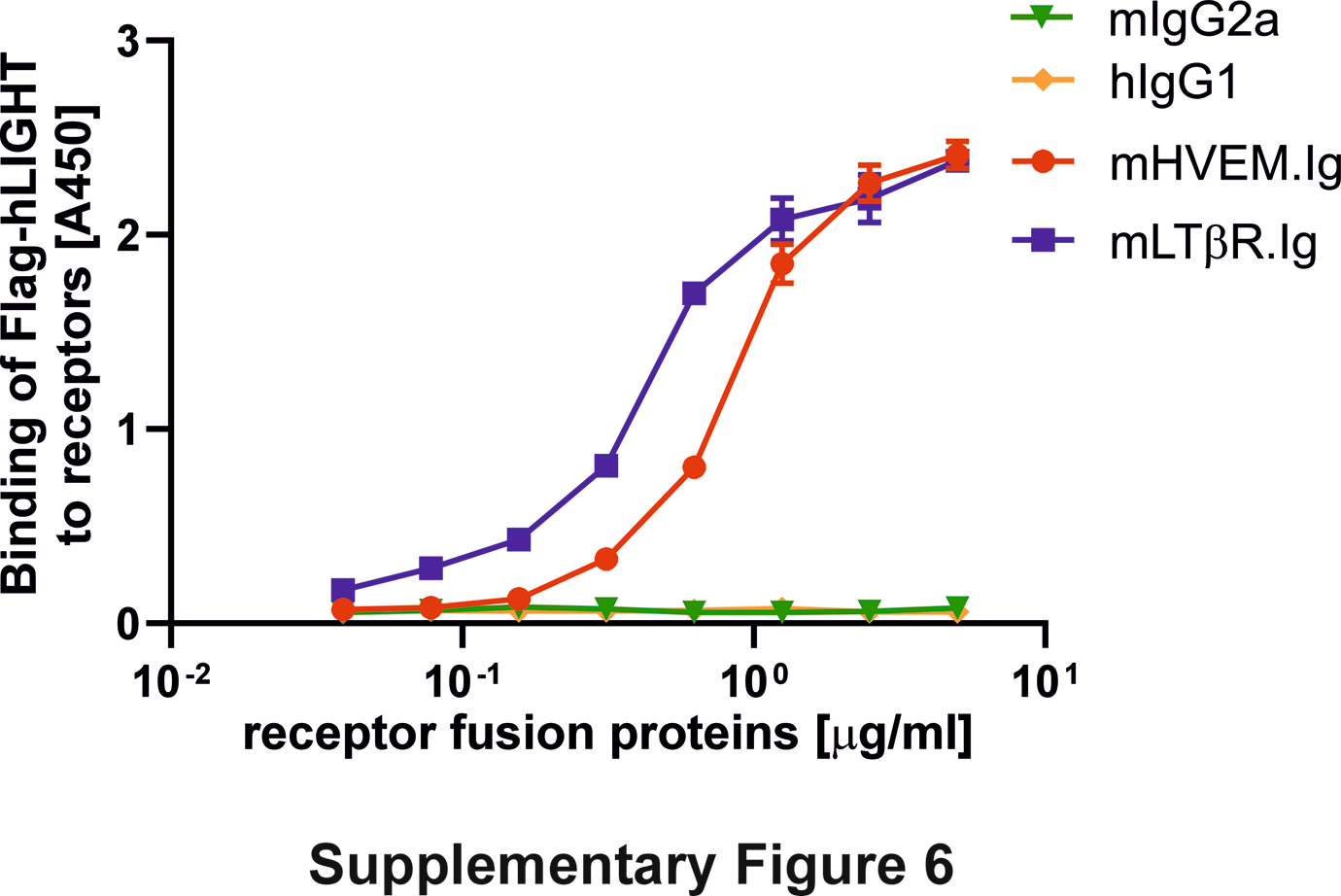

Supplement: Supplementary file 11 — (PNG 939 KB) [file 109_2025_2552_Fig11_ESM.png]

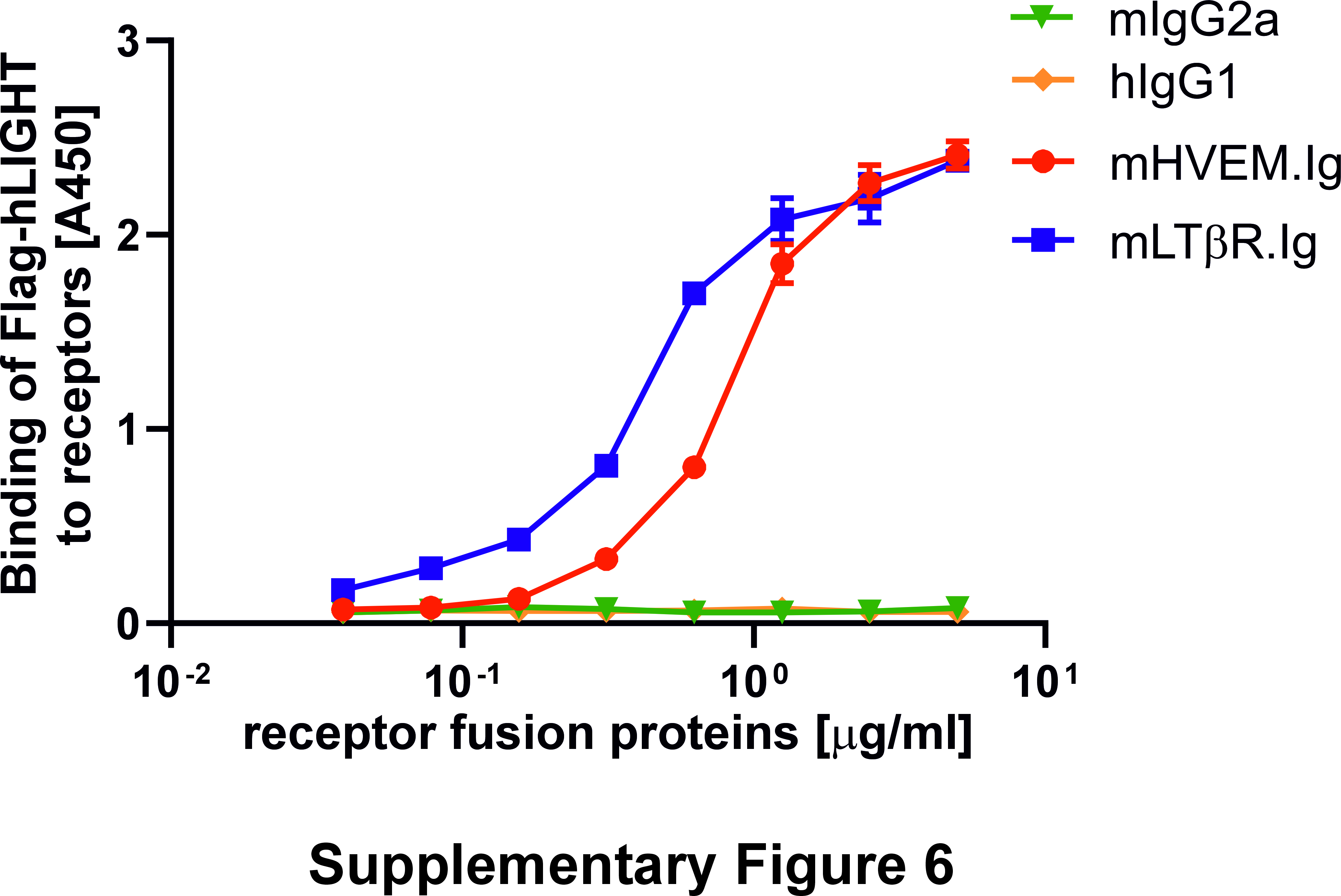

Supplement: Supplementary file 12 — Supplementary file6 (TIF 939 KB) [file 109_2025_2552_MOESM6_ESM.tif]
